# Composite outcome measures in high-impact critical care randomised controlled trials: a systematic review

**DOI:** 10.1186/s13054-024-04967-3

**Published:** 2024-05-28

**Authors:** Humphrey G. M. Walker, Alastair J. Brown, Ines P. Vaz, Rebecca Reed, Max A. Schofield, Jeffrey Shao, Vinodh B. Nanjayya, Andrew A. Udy, Toby Jeffcote

**Affiliations:** 1 Department of Critical Care, St Vincent’s Hospital, Melbourne, VIC Australia; 2 Department of Intensive Care and Hyperbaric Medicine, The Alfred Hospital, Melbourne, Australia; 3 Australian and New Zealand Intensive Care Research Centre, School of Public Health and Preventive Medicine, Monash University, Prahran, VIC Australia; 4 Department of Critical Care, University of Melbourne, Melbourne, VIC Australia; 5 Monash University, Melbourne, VIC Australia

**Keywords:** Critical care outcomes, Outcome assessment, Critical care, Randomized controlled trials as topic

## Abstract

**Background:**

The use of composite outcome measures (COM) in clinical trials is increasing. Whilst their use is associated with benefits, several limitations have been highlighted and there is limited literature exploring their use within critical care. The primary aim of this study was to evaluate the use of COM in high-impact critical care trials, and compare study parameters (including sample size, statistical significance, and consistency of effect estimates) in trials using composite versus non-composite outcomes.

**Methods:**

A systematic review of 16 high-impact journals was conducted. Randomised controlled trials published between 2012 and 2022 reporting a patient important outcome and involving critical care patients, were included.

**Results:**

8271 trials were screened, and 194 included. 39.1% of all trials used a COM and this increased over time. Of those using a COM, only 52.6% explicitly described the outcome as composite. The median number of components was 2 (IQR 2–3). Trials using a COM recruited fewer participants (409 (198.8–851.5) vs 584 (300–1566, *p* = 0.004), and their use was not associated with increased rates of statistical significance (19.7% vs 17.8%, *p* = 0.380). Predicted effect sizes were overestimated in all but 6 trials. For studies using a COM the effect estimates were consistent across all components in 43.4% of trials. 93% of COM included components that were not patient important.

**Conclusions:**

COM are increasingly used in critical care trials; however effect estimates are frequently inconsistent across COM components confounding outcome interpretations. The use of COM was associated with smaller sample sizes, and no increased likelihood of statistically significant results. Many of the limitations inherent to the use of COM are relevant to critical care research.

**Supplementary Information:**

The online version contains supplementary material available at 10.1186/s13054-024-04967-3.

## Background

Randomised controlled trials (RCT) are the gold standard by which clinicians assess current and emerging treatments in critical care medicine [1]. Unfortunately, RCTs are associated with significant financial and opportunity costs [2] and frequently do not identify statistically significant differences in outcomes for a given treatment strategy [3]. These issues are particularly marked when mortality is used as the primary outcome measure [4–6].

The selection of appropriate outcomes for critical care trials is important if they are to effectively guide clinical decision making. Trial outcomes need to be meaningful to patients, enable efficient trial design and minimise the risk of missing important treatment effects.

Composite outcome measures (COM) combine multiple clinical events (≥ 2 component outcomes) into a single outcome [7]. They are designed to capture a greater number of outcome events, and thus increase a trial’s ability to demonstrate a statistically significant treatment effect [8]. Other proposed methodological benefits include reduced sample size requirements, avoidance of the need to choose a single primary outcome, reduced use of multiple statistical comparisons [9, 10] and to account for competing risks [11]. However, significant limitations have been identified regarding the implementation [11, 12] and interpretation [8, 10] of COM. A major concern is a lack of consistency of the individual component event rates, resulting in the overall event rate being driven by individual components of the COM. This imbalance risks the creation of a “misleading impression of the impact of treatment” [13, 14]. Additionally, the outcome component driving the overall event rate may not be patient important [15].

COM are increasingly used in the design and implementation of clinical trials, and their use has been evaluated in several medical specialities [16, 17]. Within critical care, COM, such as ventilator free days (VFD), are widely used. There is also a trend towards the analysis [18] or reanalysis [19, 20] of trial data using more complex COM. However, their use is occurring in the setting of a paucity of literature evaluating the validity of COM use in critical care research.

Given the increasing utilisation of COM, the trend towards increasingly complex COM and the implications for trial validity, we performed a systematic review to summarise the use and reporting of COM in high-impact critical care RCTs. We focused specifically on COM that include patient important outcomes as there is increasing recognition that trial outcomes should be relevant to patients [21]. Our primary aim was to quantify COM use in this population, and compare study parameters (including sample size, statistical significance, and consistency of effect estimates) between trials using composite and non-composite outcomes.

## Methods

### Protocol

Methods for inclusion and analysis of studies in this systematic review were pre-specified in a protocol developed in accordance with the most recent Preferred Reporting Items for Systematic Reviews and Meta-analyses (PRISMA) [22] guidelines. This protocol was prospectively registered with PROSPERO (CRD42022380606). No funding for this study was obtained.

### Search strategy

The full search strategy is described in the online data supplement and used the following databases: Ovid MEDLINE, PubMed and the Cochrane Library. RCTs published from 1st August 2012 until 31st December 2022 were included. The reference sections of selected trials from preliminary searches were reviewed to guide the refinement of search strategies.

### Selection criteria

The titles and abstracts were independently screened by two of three reviewers (HW, AB, IV) and scored against prespecified eligibility criteria. Disagreements were resolved by a third senior reviewer (TJ). All trials deemed eligible underwent full text review prior to data extraction to ensure inclusion criteria were met*.*

We included RCTs that were conducted in the critical care population, published in the English language in sixteen high impact factor journals. Journal selection was based on a previous review by Harhay et al. [5]. The primary outcome or at least one component of the COM had to be a patient important outcome as defined by Gaudry et al. [23]: “mortality at any time and/or quality of life, functional/cognitive/neurological outcomes assessed after ICU discharge”.

Exclusion criteria included non randomised controlled trials, involving those < 18 years old or any trial that did not include a patient important outcome. The full inclusion and exclusion criteria, alongside the journals selected are published in the online data supplement (Table S1).

### Data collection and risk of bias

All selected trials were independently reviewed by two of six reviewers (HW, AB, IV, MS, JS, RR). Risk of bias analysis was performed using The Cochrane Risk of Bias 2 (RoB2) tool [24]. Data were extracted and recorded using standardised data forms on a web-based collaboration software platform (Covidence) [25]. The data forms were designed prior to the commencement of data collection. Discrepancies in risk of bias score, methodological classification or other data points were identified by the Covidence system and were resolved in consultation with a third senior reviewer (TJ, AU, VN). No missing data were imputed.

### Definitions of outcomes

Outcome measures were categorised as follows:

- *Categorical composites* in which patients were defined as having the primary outcome if they met pre-defined criteria for one or more of the discrete components.
- *Continuous composites* included any outcome that measured duration of organ support or disease process (e.g. VFD).
- *Non-COM studies* consisted of trials using:
  - *A single binary outcome* e.g. mortality
  - *Other* included those single outcomes that did not fit into the above categories. They principally measured patient performance within a single domain (such as Modified Rankin Scale or Glasgow Outcome Score as a measure of functional status)

### Outcomes and data synthesis

Each trial was primarily categorised as a COM or non-COM trial. COM trials were then sub-categorised according to the classification above. When classifying components of a COM, all potential possibilities of meeting the outcome were included as individual components. For example, the COM “Days Alive and Free of Life Support” included 4 potential outcomes: mortality, duration of mechanical ventilation, circulatory support, and renal replacement therapy respectively. Trials with co-primary outcomes were included. If a co-primary outcome consisted of both a non-COM and a COM e.g. Kawazoe et al. [26], then it was classified as a COM. Trials were also included if utilising both patient and non-patient important co-primary outcomes e.g. Zarbock et al. [27].

For comparison of achieved effect sizes, the difference between predicted and observed effect size was calculated. Predicted relative effect estimates were calculated to compare the predicted magnitude of effect used for power calculations. Methods for calculating the event rate gap are described in the online data supplement. Trials with more than two arms, 2 × 2 factorial designs or studies with co-primary outcomes were not included in event rate and effect size analyses. Data for comparison of event rates or effect sizes, when presented as a range of values or without reference to an absolute risk difference were excluded.

### Statistical analysis

Continuous data are presented as median (interquartile range) and mean (standard deviation) where appropriate. Categorical data are presented as frequency (%). The analysis was primarily descriptive, albeit where comparisons were made, the Mann–Whitney U Test and Fisher’s Exact test have been used, as indicated. The analysis of COM use over time was conducted using the Mann Kendall trend test. To calculate the predicted relative effect estimate, the predicted effect sizes were converted into relative risk estimates by dividing the predicted absolute risk reduction by the expected event rate in the control group. The same principle was used for continuous outcomes (e.g. if an effect size of 2 VFD was predicted with a control rate of 8, then this was a 25% relative difference). These have been separated due to the differences in data and scale used (e.g. VFD and % reduction in mortality).

We conducted a sensitivity analysis in which we included ordinal outcome scales such as mRS (modified Rankin Scale) as COM, and also removed them from the analysis all together.

Significance has been assumed with a *p*-value < 0.05 and no adjustment for multiplicity was made. All statistical analyses were conducted using GraphPad Prism 10.0.3 [28].

## Results

**Fig. 1:**
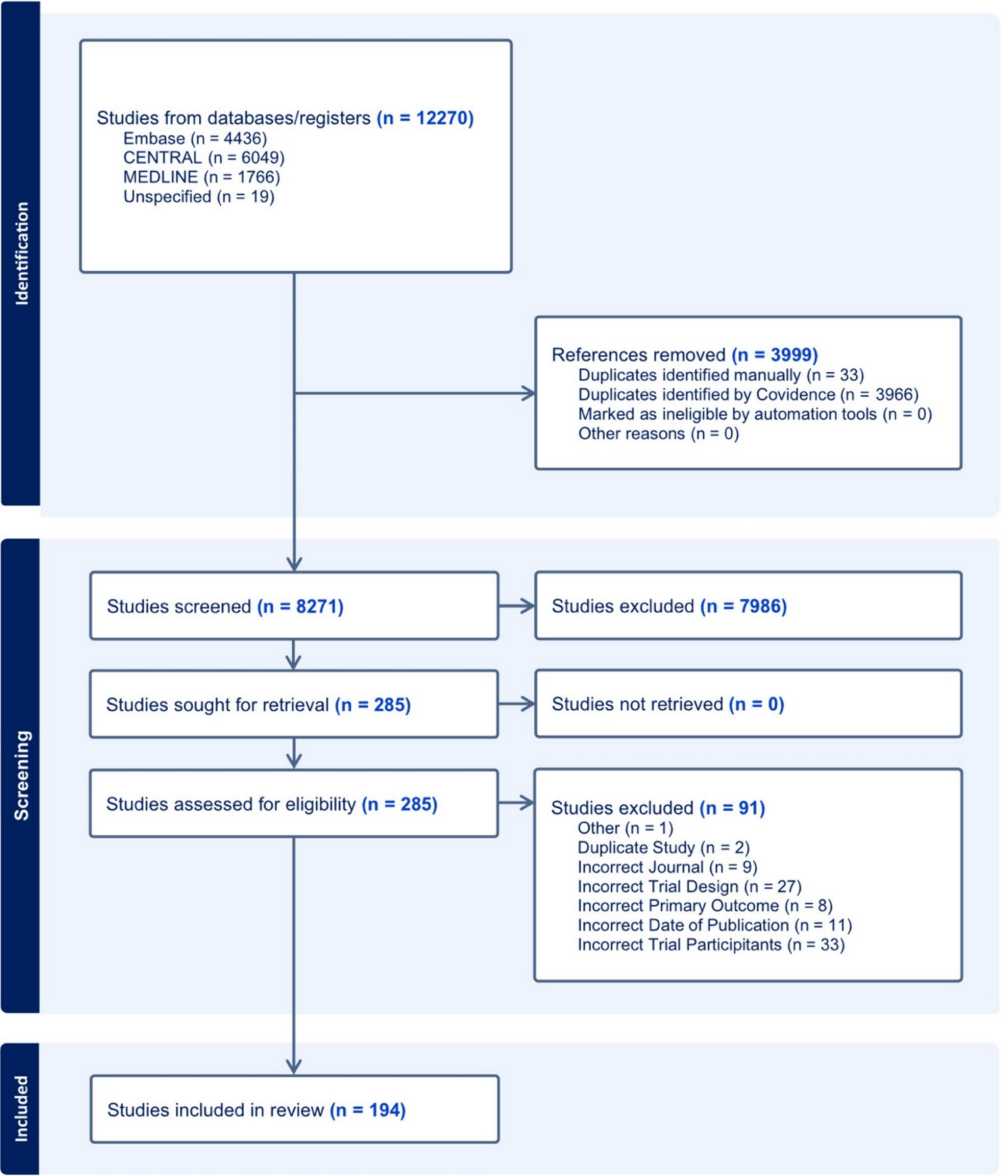
Showing PRISMA flow diagram of trial selection

12,270 trials were identified following the database search and after removal of duplicates, 8271 records underwent title and abstract screening. 194 trials were included in the final analysis. This is shown in Fig. 1. A reference list is included in the online data supplement.

### Trial characteristics

Of the 194 trials included in the analysis, 76 (39.1%) RCTs were classified as using a COM for the primary outcome. For trials using a non-COM, 101 assessed mortality (or survival) at various time points as a primary outcome. The basic characteristics of the included trials are outlined in Table 1. Figures S1 and S2 in the online data supplement show all populations and interventions studied in the included trials.

**Table 1:** Showing the demographics and characteristics of included studies

Variable (number of trials reporting^a^)	COM (n = 76)	Non-COM (n = 118)
Mean Age, years (SD) (n = 117)	61.4 (5.3)	60 (6.8)
Male Sex %, median (IQR) (n = 192)	64.4 (57.9–70.9)	60.9 (56.8–65.8)
Mechanical ventilation %, median (IQR) (n = 119)	82.2 (34.5–100)	82.2 (61.5–100)
Vasoactive use %, median (IQR) (n = 79)	54.8 (20.9–77.4)	70.3 (46.7–95.5)
Mean APACHE II score (SD) (n = 48)	20.8 (4.3)	22.3 (4.3)
Mean SOFA score (SD) (n = 40)	9 (2.6)	9.9 (2.3)
**Population studied, n (% of trials)**		
ARDS	6 (7.9)	9 (7.6)
COVID-19	17 (22.4)	7 (5.9)
Cardiac arrest	4 (5.3)	10 (8.5)
Sepsis	16 (21.1)	33 (28)
Cardiac surgical	7 (9.2)	2 (1.7)
General ICU population^b^	16 (21.1)	36 (30.5)
**Intervention studied, n (% of trials)**		
Ventilation/oxygenation	13 (17.1)	14 (11.9)
Vasoactive medications	5 (6.6)	4 (3.4)
Blood products	3 (3.9)	8 (6.8)
Cooling	2 (2.6)	8 (6.8)
Fluid	3 (3.9)	8 (6.8)
Nutrition	1 (1.3)	7 (5.9)
Other drugs^c^	36 (47.4)	32 (27.1)

^a^The number of trials reporting reflects the number of trials from which this data was extracted. The maximum was 194. The data for trial characteristics was only analysed if the data was presented as a mean

^b^*“*General ICU Population*”* was selected when the intervention being assessed did not fit one particular group of patients (e.g. O2 targets in all ventilated patients*)*

^c^*“*Other *D*rugs*”* include all those drug therapies not listed here (i.e. non-vasoactive medications, blood products or fluid). Not all populations or interventions studied are included in this table (see Figures S1 and S2 in the online data supplement)

### COM versus non-COM

**Fig. 2:**
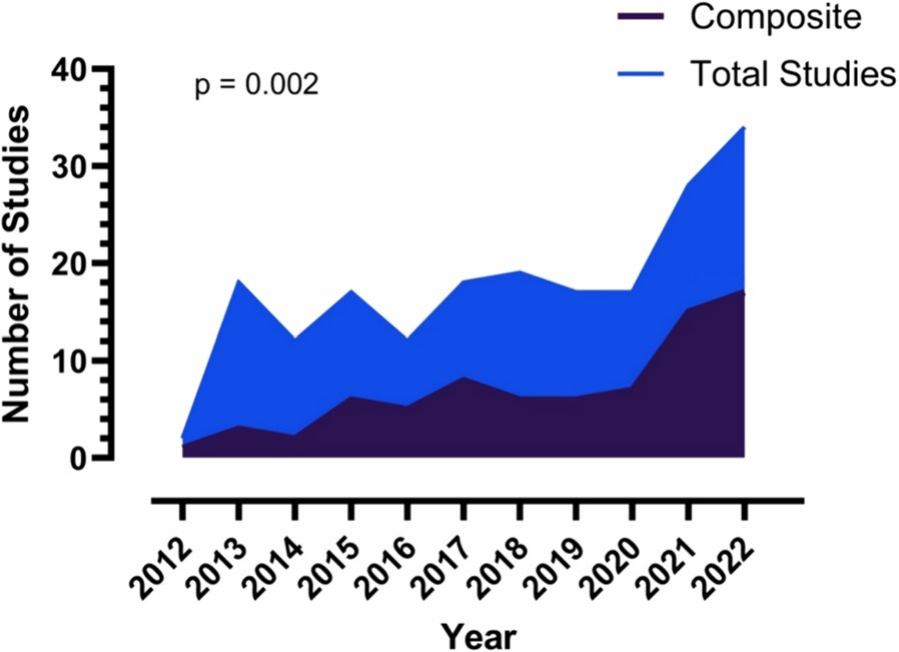
Showing the trend of COM use as a proportion of all trials included in the systematic review

The use of COMs in high-impact critical care RCTs has significantly increased since 2012 (*p* = 0.002 for trend over time, Fig. 2). Comparing RCTs using a COM vs non-COM respectively, significantly fewer patients were enrolled [409 (198.8–851.5) vs 584 (300–1566) patients, *p* = 0.004] and the median number of sites was lower [12 (2–35) vs 31 (11.5–50.5) sites, *p* = 0.009]. The median reported power for COM and non-COM trials were similar at 80 (IQR 80–85) % and 80 (IQR 80–90) % respectively. This is shown in Table 2 (and Fig. S3 in the online data supplement).

**Table 2:** Showing selected differences in trial characteristics and data presentation between (i) trials using a COM and a non-COM and (ii) trials using a categorical COM and continuous COM

	Non-COM (n = 118)	All COM (n = 76)	Categorical COM (n = 31)	Continuous COM (n = 45)
Median number of sites, n (IQR)	31 (11.5–50.5)	12 (2–35)	4 (1–16)	18 (6.5–42.5)^a^
	Comparing non-COM versus COM *p* = 0.009 Comparing categorical versus Continuous *p* = 0.001			
Median number of patients, n (IQR)	584 (300–1566)	409 (198.8–851.5)	407 (192–802)	421 (201.5–881.5)
	Comparing non-COM versus COM *p* = 0.004 Comparing categorical versus continuous *p* = 0.881			
Median number of components, n (IQR)	NA	2 (2–3)	3 (2–6)	2 (2–2.5)
	Comparing categorical versus continuous *p* < 0.001			
Trial Power, median (IQR)	80 (80–85)	80 (80–90)	NA	NA
	Comparing Non-COM versus COM *p* = 0.052			
Statistically significant, n (%)	21 (17.8)	15 (19.7)	8 (25.8)	7 (15.6)
	Comparing Non-COM versus COM *p* = 0.85 Comparing Categorical vs Continuous p = 0.380			
Effect estimates consistent across all components of COM, n (%)^b^	NA	33 (56.9)	15 (48.4)	18 (66.7)
	Comparing categorical versus continuous *p* = 0.192			
Manuscripts in which outcome is stated as a COM, n (%)	NA	40 (52.6)	26 (83.9)	14 (31.1)
	Comparing categorical versus continuous *p* < 0.001			
Each component presented separately in the table of results, n (%)	NA	49 (64.5)	25 (80.7)	24 (53.3)
	Comparing categorical versus continuous *p* = 0.017			
Each component of COM clearly defined n (%)	NA	75 (98.7)	32 (100)	31 (97.7)
	Comparing categorical versus continuous *p* > 0.99			

^a^1 trial using a continuous COM did not report data for number of trial sites

^b^It was not possible to ascertain whether all effect estimates were consistent in 18 trials (all continuous) therefore for all COM n = 58, and for continuous COM n = 27

Of note, a significant proportion of trials using a COM (n = 17, 22.4%) investigated COVID-19. Fewer (n = 7, 5.9%) non-COM trials investigated this disease. 40/76 (52.6%) of those using a COM were explicitly described as a COM in the manuscript. 49 (64.5%) had all components reported in the table of results. There was no difference in Risk of Bias assessment scores between COM and non-COM trials (see Fig. S4 in the online data supplement).

**Fig. 3:**
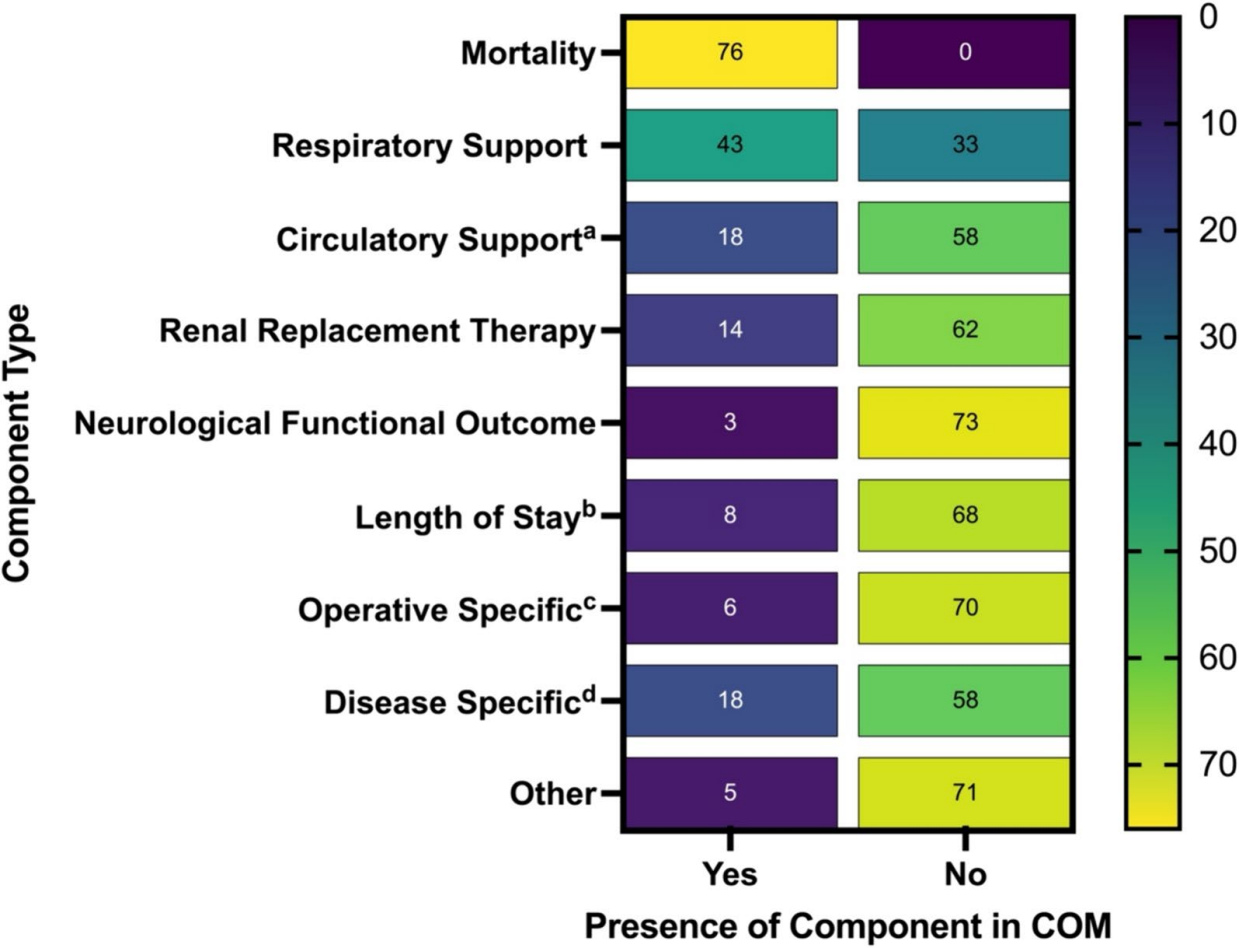
Showing a heatmap of all the components used in COMs within this systematic review. ^a^Circulatory support includes extracorporeal membrane oxygenation (ECMO). ^b^Includes outcomes assessing both hospital and ICU length of stay. ^c^Operative specific components include outcomes such as re-operation. ^d^Disease specific components includes development of specific diseases or syndromes. For example, myocardial infarction (MI), acute kidney injury (AKI), Acute Respiratory Distress Syndrome (ARDS) or venous thromboembolic event (VTE)

The median number of components was 2 (2–3), and 5 (6.6%) trials utilised a COM composed entirely of patient important components. All of the COM included mortality as a component of the composite outcome. Other component types utilised are shown in Fig. 3. A comprehensive list of all the components utilised in the included trials is provide in the online data supplement (Table S2).

### Categorical versus continuous COM

31 (40.8%) of the COM were categorical, with the remaining 45 (59.2%) being continuous. Of the continuous COM, 36 (80.0%) were organ support free days (OSFD). Of these, 7 (15.6%) scored death as -1. Identification within the trial manuscript that a COM was being utilised occurred less frequently in the continuous COM group (83.9% vs 31.1%, *p* < 0.001). This is shown in Table 2.

### Consistency of effect estimates and effect sizes

In trials using a COM, it was only possible to assess whether point effect estimates were consistent in 58 (76.3%) of trials with the information provided. Point effect estimates were consistent (e.g. all in favour or not in favour of intervention) across all components in 33 (56.9%) of trials. In the remaining trials, point estimates were not consistent (n = 16 categorical and n = 9 continuous). There was no apparent difference between trials using a categorical or continuous COM [15/31 (48.4%) vs 18/45 (66.7%), *p* = 0.192]. Mortality was the component with the highest event rate in 15/31 (48.4%) of trials using a categorical COM.

**Fig. 4:**
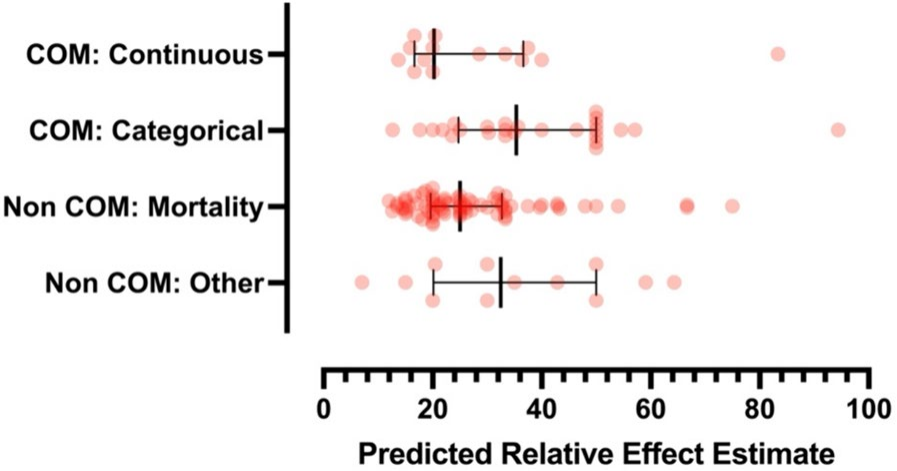
Showing the predicted relative effect estimates for COM and Non-COM studies. Error bars are median and IQR

The predicted relative effect estimates are shown in Fig. 4. For trials using a categorical COM this was 35.4 (IQR 24.8–50.0), and for trials using a continuous COM this was 20.2 (IQR 16.7–36.7). For non-COM trials that assessed mortality this was 25.0 (IQR 19.6–32.7) and for other non-COM trials this was 38.9 (IQR 22.5–56.8). Predicted relative effect estimates were significantly greater for categorical COM compared to non-COM trials assessing mortality (*p* < 0.001). Most trials had predicted effect sizes greater than the actual effect size, with only 6 trials having an effect size larger than predicted**.** This is shown in Fig. S5 in the online data supplement. The event rate gap was similar between COM and Non-COM trials (Table S3 and Fig. S6 in the online data supplement).

### Reported statistical significance

There was no difference in the frequency of reported statistical significance between COM and non-COM trials [17/56 (19.7%) vs 21/118 (17.8%), *p* = 0.85]. Rates of statistical significance did not vary between categorical COM and continuous COM [8/31 (25.8%) vs 7/45 (15.6%), *p* = 0.38].

### Sensitivity analyses

19 trials used ordinal outcomes. Results in all sensitivity analyses were unchanged with respect to number of sites, number of participants, trial power and rates of statistical significance (Tables S5–S8 in the online data supplement).

## Discussion

We performed a systematic review of critical care trials published in high impact journals with a specific focus on COM. We assessed the frequency of use and reporting of COM in the relevant studies. We documented the different forms of COM, the consistency of treatment effect between individual components of the composites and the proportion of patient centred outcomes within these composites. Lastly, we compared the accuracy of effect size calculations and frequency of statistically significant results between COM and non-COM studies.

The recently published CONSORT-Outcomes 2022 extension [7] calls for all components of a COM to be reported in order to clarify the interpretation of trial findings. Only 65% of trials identified in our systematic review had complete reporting of all individual components in the manuscript. Additionally, only 50% of the trials explicitly acknowledged the use of a COM in the published manuscript. There was a notable lack of acknowledgement of the composite nature of continuous COM such as organ support free days, a finding that is consistent with previous literature [29]. The median number of components for all trials with a COM was 2 (IQR 2–3) and the maximum number of components was 21 [30]**.** This is important as increasing components will complicate the interpretation of which components are driving any treatment effect identified by clinical trials. The number of trials using a COM composed entirely of patient important outcomes was low (7%). Treatment effects were therefore partly determined by non-patient important outcomes (e.g. asymptomatic DVT, acute kidney injury, worsening oxygenation, radiographic progression) in 93% of trials. While we recognise the clinical validity of a range of non-patient important outcomes, we would argue that these should be analysed in a way that accounts for the varying importance of specific components within a COM. One analytical method that allows for the prioritisation of outcomes within a single COM may be the Win Ratio [31] which has been used recently in the re-analysis of several large critical care trials and may mitigate the challenge of the competing risk of death and other components of composite outcomes [19, 20].

Despite the well documented challenges associated with their use [29, 32] continuous COM, and particularly OSFDs, were used widely (60% and 50% respectively). One specific issue with these forms of outcome measure is inconsistency of mortality scoring within the continuous COM. Our review revealed that both mortality and ongoing dependence on organ support were scored as zero in approximately 80% of studies, whereas in a further 20%, death was scored as minus one and ongoing dependence on support as zero. This heterogeneity has been previously documented [33] and is a significant barrier to study comparisons and meta-analyses. A widely accepted standard for the calculation of failure free days has not yet been established but could reduce methodological heterogeneity [34].

48% of categorical COM demonstrated consistency of treatment effect across COM components. Given the complexity of physiological derangements in critically ill patients this finding is not unexpected. There is, however, an argument for trialists to demonstrate biological plausibility for the components of a COM. Further, the explicit recognition of trial outcomes that are based on conflicting treatment effects or entirely driven by a single component of a COM should be encouraged.

Potential benefits of COM include increased power and/or reductions in sample size requirements [8, 12]. These may be particularly valuable in critical care trials where overestimation of effect size has been consistently demonstrated [5, 35]. However, our review revealed that only 1 trial utilising a COM had an effect size greater than predicted [36]. Greater predicted relative effect estimates for categorical COM in comparison to non-COM trials with mortality as the primary outcome was shown, and median study power for both COM and non-COM studies were similar, implying that in critical care trials COM are employed primarily to reduce sample size requirements rather than increase statistical power. In some settings (such as the recent COVID-19 pandemic) smaller sample sizes may facilitate rapid assessment of interventions. Indeed, our findings indicate increased use of continuous COM during the COVID-19 pandemic. We would caution however, that the use of COM to reduce sample size necessitates increased scrutiny of the components of the COM, the heterogeneity of component treatment effects and the degree of patient centredness. We would also argue for pre-trial documentation of COM components, expected treatment effects and the specification of what the investigators consider a minimum clinically important difference (MCID) [37] to determine if a particular result represents a genuine treatment effect or a type 2 error.

The use of COM has been associated with a trend towards increased rates of statistical significance in other specialties such as cardiology [38]. In our review there was no difference in rates of statistical significance between COM and non-COM trials. Additionally, the rate of statistical significance in our review (18.5%), is lower than previously reported for critical care trials [3, 5]. It is noteworthy though, that previous reviews were not restricted to patient important outcomes. The observation that rates of statistical significance for clinical trials utilising COM differ between different medical specialties may reflect differences in the disease states investigated. A significant sub-set of the diseases investigated in critical care trials e.g. traumatic brain injury, acute respiratory distress syndrome, can be characterised as physiological syndromes rather than diseases with a clear pathophysiology such as acute myocardial ischaemia. The heterogeneity of critical care syndromes is increasingly recognised as a confounding factor in critical care trials [39]. A COM which reduces sample size requirements for a predicted treatment effect but fails to capture this heterogeneity will not lead to increased rates of statistically significant trials. While careful patient selection and cohort enrichment are key factors in improving trial efficiency in critical care, the flexibility of COM represents an opportunity for significantly improved critical care trial design. A thoughtfully designed COM that can represent the heterogenous nature of a critical care syndrome may be able to successfully demonstrate treatment effects with smaller sample sizes and the use of fewer resources. Recommendations based on the findings from this systematic review regarding the use of COM in critical care trials are made in Table 3.

**Table 3:** Summarising issues identified in this systematic review and suggested recommendations regarding the future use of COM in critical care

	Issue identified	Suggested recommendation
Design	Component of COM occurring most frequently not patient important	The importance of both clinician and patient engagement in the design of COM relevant to critical care
	Point effect estimates for each component not consistent in direction of effect	The significance of an inconsistency of treatment effects between components of a COM should be addressed in the statistical analysis plan. Further work looking into the utility of more complex COM (e.g. hierarchical composite endpoints such as the Win Ratio) should be conducted as this provides a method for the integration of multiple levels of outcomes
	Predicted effect estimate greater than achieved, alongside tendency to reduce sample size, as opposed to increase statistical power	Effect size calculations for trials using a COM should be based as much as possible on available data. Pre-trial specification of what constitutes a minimally clinically important difference should be documented
Reporting	Outcome not documented as a COM within the manuscript	We recommend an increased awareness around the use of COM including continuous COM such as organ support free days. If a COM is used for a clinical trial, it should be recognised and reported as such
	Lack of presentation of all individual components of the COM in the results	All individual components of the COM should be reported in a manner to allow clinicians to easily interpret which components are driving the overall effect. Additionally, within the constraints of ethical and data protection principles, raw individual data should be made available to increase cross-trial usability of COM results

## Strengths and limitations

To our knowledge this is the first systematic review of COM use in the critical care RCTs. We have utilised a robust and reproducible search strategy and a study selection methodology based on prior literature designed to assess the outcomes and characteristics of ICU based RCTs [5]. We have sought to distinguish between continuous and categorical COM, document the reporting of COM, assess the validity of sample size calculation and the frequency of statistically significant results. Given their increasing use, the lack of literature exploring their characteristics and the advent of more complex COM we submit that this is a timely and useful review of the literature.

Limitations of the review include the focus on 16 high impact critical care journals. There will undoubtedly be critical care trials using patient focused COM that are not captured by this review. However, we feel that practice change is predominantly based on larger trials published in high impact journals so a focus on this category of evidence is justified. Our review also omits studies utilising COM without any patient centred components. We feel that our use of a recognised definition of patient centred outcomes [23] and the increasing importance of this concept [40] adds weight to the validity of this approach. The omission of ordinal scales such as the modified Rankin scale or Glasgow outcome scale from our definition of COM could be considered inconsistent with our inclusion of continuous COM such as ventilator free days. We acknowledge the challenges and nuances associated with classification of these outcomes. Our decision to classify them as non-COM for our primary statistical analyses results from finding no examples of these scales being described as COM within the literature, which contrasts to OSFDs [29]. Additionally, it could be suggested that all levels of ordinal scales assessing neurological outcome fall within the single domain of function [41]. In recognition of the ambiguity surrounding their classification we performed multiple sensitivity analyses. The results of these analyses do not materially change our conclusions. Additionally, the sensitivity analyses of these scales reveal heterogeneity in terms of their application and analysis, a finding that has previously been described [42, 43].

## Conclusion

In high-impact critical care RCTs assessing patient important outcomes, COM are used frequently. The use of COM has increased over time. The inherent limitations of COM identified in trials from related medical specialties, are also relevant to critical care. Many trials using a COM did not demonstrate consistency across all components of the COM and 93% of COM included non patient-important components. The primary methodological benefit of a COM is a reduction in sample size. However, their use does not lead to increased rates of statistically significant results in critical care trials and predicted effect sizes remain grossly over optimistic. Further work to improve the use of COM in critical care should focus on the design and validation of COM that include patient important outcomes and effectively represent the heterogeneity of the pathologies studied in the critical care literature.

### Supplementary Information

Supplementary Information

## Data Availability

The datasets used and analysed during the current study are available from the corresponding author on reasonable request.

## Abbreviations

RCT: Randomised controlled trial
COM: Composite outcome measure
Non-COM: Non-composite outcome measure
VFD: Ventilator free days
ECMO: Extracorporeal membrane oxygenation
MI: Myocardial infarction
AKI: Acute kidney injury
ARDS: Acute respiratory distress syndrome
VTE: Venous thromboembolic event
OSFD: Organ support free days
mRS: Modified rankin scale
GOS-E: Glasgow outcome score extended
