# Supplementary material for: Composite outcome measures in high-impact critical care randomised controlled trials: a systematic review
Source: Crit Care. 2024 May 28;28:184. doi: 10.1186/s13054-024-04967-3 (PMC11134769; doi:10.1186/s13054-024-04967-3)
Supplement: Supplementary file 1 — Supplementary Information [file 13054_2024_4967_MOESM1_ESM.docx]

**Online Data Supplement**

**Contents**

1. Search Strategy
2. List of included references
3. Table S1 Inclusion and Exclusion Criteria
4. Table S2 showing all COM used within the systematic review
5. Figure S1 showing all the populations studied in the trials included
6. Figure S2 showing all the interventions studied in the trials included
7. Figure S3 showing overall Risk of Bias for COM and non-COM trials
8. Figure S4 showing study power for COM and non-COM trials
9. Figure S5 showing Figure S5 showing the predicted and achieved effect sizes for composite and non-composite studies
10. Table S3 and Figure S6 showing event rate gaps
11. Table S4 showing predicted relative effect estimates
12. Table S5 including all ordinal scales as COM
13. Table S6 including all ordinal scales as non COM
14. Table S7 excluding ordinal outcomes from the analysis all together
15. Table S8 comparing ordinal outcomes against COM
16. Table S9 showing the breakdown between favourable and unfavourable outcomes when ordinal outcomes were dichotomised

**Search Strategy**

The following is the strategy for the initial search. When the search was re-run the exact same strategy was used except the dates were extended as per the methods section.

**Ovid MEDLINE(R) <1946 to July Week 4 2022>**

**Ovid MEDLINE(R) Epub Ahead of Print <July 29, 2022>**

1 "new england journal of medicine".jn. 82394

2 jama.jn. 75537

3 "american journal of respiratory & critical care medicine".jn. 17552

4 critical care medicine.jn. 22551

5 intensive care medicine.jn. 11718

6 chest.jn. 36309

7 anesthesia & analgesia.jn. 26355

8 anesthesiology.jn. 24275

9 "annals of internal medicine".jn. 34324

10 jama internal medicine.jn. 5321

11 "british journal of anaesthesia".jn. 20145

12 canadian medical association journal.jn. 16518

13 bmj.jn. 75778

14 "journal of critical care".jn. 4143

15 "journal of trauma and acute care surgery".jn. 4715

16 lancet.jn. 139222

17 1 or 2 or 3 or 4 or 5 or 6 or 7 or 8 or 9 or 10 or 11 or 12 or 13 or 14 or 15 or 16 596857

18 critical care/ or early goal-directed therapy/ 58643

19 intensive care units/ or burn units/ or coronary care units/ or respiratory care units/ 73814

20 Critical Illness/ 36431

21 multiple organ failure/ or shock, cardiogenic/ or shock, hemorrhagic/ or shock, surgical/ or shock, traumatic/ or systemic inflammatory response syndrome/ 44365

22 resuscitation/ or cardiopulmonary resuscitation/ or respiration, artificial/ 100954

23 respiration, artificial/ or noninvasive ventilation/ or positive-pressure respiration/ or ventilator weaning/ or tracheostomy/ 81296

24 Intubation, Intratracheal/ 38819

25 Respiratory Distress Syndrome/ 23328

26 Acute Kidney Injury/ 51993

27 Continuous Renal Replacement Therapy/ 602

28 ((intensive or critical*) adj (care or ill*)).mp. 260489

29 (ICU or ITU or HDU).mp. 65088

30 ((high dependency or coronary care or burn? or respiratory support or respiratory care) adj unit*).mp. 12681

31 (shock adj1 (septic or sepsis or hem* or haem* or card* or vas* or trau*)).mp. 70169

32 resus*.mp. 87155

33 ((artific* or positive or invasive or mechanical) adj4 (ventil* or respir*)).mp. 115672

34 (trach* adj3 intubation).mp. 9372

35 (ARDS or acute resp* distress syndrome).mp. 22503

36 (AKI or acute kidney injury).mp. 60733

37 (CRRT or cont* renal replacement therapy).mp. 3231

38 18 or 19 or 20 or 21 or 22 or 23 or 24 or 25 or 26 or 27 or 28 or 29 or 30 or 31 or 32 or 33 or 34 or 35 or 36 or 37 591489

39 "randomized controlled trial".pt. 572922

40 (random$ or placebo$ or single blind$ or double blind$ or triple blind$).ti,ab. 1261013

41 (retraction of publication or retracted publication).pt. 9432

42 or/39-41 1373213

43 (animals not humans).sh. 4995002

44 ((comment or editorial or meta-analysis or practice-guideline or review or letter) not "randomized controlled trial").pt. 4807381

45 (random sampl$ or random digit$ or random effect$ or random survey or random regression).ti,ab. not "randomized controlled trial".pt. 100035

46 42 not (43 or 44 or 45) 970078

47 17 and 38 and 46 5895

48 limit 47 to english language 5895

49 limit 48 to yr="2012 -Current" 2007

50 limit 49 to ("all infant (birth to 23 months)" or "all child (0 to 18 years)" or "newborn infant (birth to 1 month)" or "infant (1 to 23 months)" or "preschool child (2 to 5 years)" or "child (6 to 12 years)" or "adolescent (13 to 18 years)") 287

51 49 not 50 1720

52 limit 51 to (case reports or comment or congress or editorial or letter or meta analysis or "systematic review") 67

53 51 not 52 1653

**Embase <1974 to 2022 July 29>**

1 "new england journal of medicine".jn. 54725

2 jama.jn. 2520

3 lancet.jn. 94378

4 "american journal of respiratory and critical care medicine".jn. 81728

5 critical care medicine.jn. 40625

6 intensive care medicine.jn. 20494

7 chest.jn. 54658

8 anesthesia analgesia.jn. 70

9 anesthesiology.jn. 23359

10 "annals of internal medicine".jn. 32030

11 jama internal medicine.jn. 6018

12 canadian medical association journal.jn. 6596

13 "british journal of anaesthesia".jn. 20482

14 bmj.jn. 2390

15 "journal of critical care".jn. 4702

16 "journal of trauma and acute care surgery".jn. 4632

17 1 or 2 or 3 or 4 or 5 or 6 or 7 or 8 or 9 or 10 or 11 or 12 or 13 or 14 or 15 or 16 449407

18 intensive care/ or artificial feeding/ or early goal-directed therapy/ or patient monitoring/ 235665

19 intensive care unit/ or burn unit/ or coronary care unit/ or medical intensive care unit/ or neurological intensive care unit/ or surgical intensive care unit/ 219702

20 critical illness/ 33803

21 shock/ or cardiogenic shock/ or hemorrhagic shock/ or hypovolemic shock/ or septic shock/ or traumatic shock/ or vasodilatory shock/ 143718

22 resuscitation/ or advanced cardiac life support/ 124036

23 artificial ventilation/ or intermittent mandatory ventilation/ or invasive ventilation/ or noninvasive ventilation/ or positive pressure ventilation/ or prone ventilation/ or ventilator weaning/ 183099

24 endotracheal intubation/ or rapid sequence induction/ 57211

25 adult respiratory distress syndrome/ 50211

26 acute kidney failure/ 107664

27 continuous renal replacement therapy/ or continuous hemodiafiltration/ or continuous hemodialysis/ or continuous hemofiltration/ 12298

28 ((intensive or critical*) adj (care or ill*)).mp. 500719

29 (ICU or ITU or HDU).mp. 154018

30 ((high dependency or coronary care or burn? or respiratory support or respiratory care) adj unit*).mp. 21960

31 (shock adj1 (septic or sepsis or hem* or haem* or card* or vas* or trau*)).mp. 126890

32 resus*.mp. 177561

33 ((artific* or positive or invasive or mechanical) adj4 (ventil* or respir*)).mp. 218991

34 (trach* adj3 intubation).mp. 14868

35 (ARDS or acute resp* distress syndrome).mp. 41647

36 (AKI or acute kidney injury).mp. 60177

37 (CRRT or cont* renal replacement therapy).mp. 9638

38 18 or 19 or 20 or 21 or 22 or 23 or 24 or 25 or 26 or 27 or 28 or 29 or 30 or 31 or 32 or 33 or 34 or 35 or 36 or 37 1153299

39 (random$ or placebo$ or single blind$ or double blind$ or triple blind$).ti,ab. 1955748

40 RETRACTED ARTICLE/ 13083

41 or/39-40 1968303

42 (animal$ not human$).sh,hw. 4666998

43 (book or conference paper or editorial or letter or review).pt. not exp randomized controlled trial/ 5612919

44 (random sampl$ or random digit$ or random effect$ or random survey or random regression).ti,ab. not exp randomized controlled trial/ 144521

45 41 not (42 or 43 or 44) 1499885

46 17 and 38 and 45 8744

47 limit 46 to english language 8733

48 limit 47 to yr="2012 -Current" 4507

49 limit 48 to (embryo <first trimester> or infant <to one year> or child <unspecified age> or preschool child <1 to 6 years> or school child <7 to 12 years> or adolescent <13 to 17 years>) 364

50 48 not 49 4143

51 limit 50 to (conference abstracts and evidence based medicine and (conference abstract or editorial or letter)) 4

52 50 not 51 4139

53 limit 52 to medline 100

54 52 not 53 4039

**CENTRAL**

Date Run: 01/08/2022 07:34:42

Comment:

ID Search Hits

#1 (new england journal of medicine):so OR (jama):so OR (lancet):so OR (american journal of respiratory and critical care medicine):so OR (critical care medicine):so (Word variations have been searched) 37146

#2 (intensive care medicine):so OR (chest):so OR (anesthesia analgesia):so OR (anesthesiology):so OR (annals of internal medicine):so (Word variations have been searched) 25708

#3 (jama internal medicine):so OR (canadian medical association journal):so OR (british journal of anaesthesia):so OR (bmj):so OR (journal of critical care):so (Word variations have been searched) 22844

#4 (journal of trauma and acute care surgery):so (Word variations have been searched) 186

#5 #1 or #2 or #3 or #4 77307

#6 ("intensive care unit"):ti,ab,kw OR ("intensive care"):ti,ab,kw OR ("critical care unit"):ti,ab,kw OR ("high dependency unit"):ti,ab,kw OR ("high-dependency unit"):ti,ab,kw (Word variations have been searched) 27239

#7 ("burn unit"):ti,ab,kw OR ("coronary care unit"):ti,ab,kw OR ("respiratory care unit"):ti,ab,kw OR ("ICU"):ti,ab,kw OR ("ITU"):ti,ab,kw (Word variations have been searched) 17866

#8 ("HDU"):ti,ab,kw OR ("septic shock"):ti,ab,kw OR ("cardiogenic shock"):ti,ab,kw OR ("haemorrhagic shock"):ti,ab,kw OR ("traumatic shock"):ti,ab,kw (Word variations have been searched) 4900

#9 (resus*):ti,ab,kw OR ("artificial ventilation"):ti,ab,kw OR ("mechanical ventilation"):ti,ab,kw OR ("positive pressure ventilation"):ti,ab,kw OR ("positive pressure respiration"):ti,ab,kw (Word variations have been searched) 26666

#10 ("artificial respiration"):ti,ab,kw OR ("tracheal intubation"):ti,ab,kw OR ("acute respiratory distress syndrome"):ti,ab,kw OR ("acute renal failure"):ti,ab,kw OR ("CRRT"):ti,ab,kw (Word variations have been searched) 8527

#11 ("continuous renal replacement therapy"):ti,ab,kw (Word variations have been searched) 501

#12 #6 or #7 or #8 or #9 or #10 or #11 61446

#13 #5 and #12 with Publication Year from 2012 to 2022, in Trials 5852

**References of Included Studies**

Allingstrup MJ, Kondrup J, Wiis J, Claudius C, Pedersen UG, Hein-Rasmussen R, et al. Early goal-directed nutrition versus standard of care in adult intensive care patients: the single-centre, randomised, outcome assessor-blinded EAT-ICU trial. Intensive Care Med 2017; 43:1637-1647.

Andersen-Ranberg NC, Poulsen LM, Perner A, Wetterslev J, Estrup S, Hästbacka J, et al. Haloperidol for the Treatment of Delirium in ICU Patients. N Engl J Med. 2022; 387:2425-2435.

Andrews PJ, Sinclair HL, Rodriguez A, Harris BA, Battison CG, Rhodes JK, et al. Hypothermia for Intracranial Hypertension after Traumatic Brain Injury. N Engl J Med 2015; 373:2403-12..

Angus DC, Derde L, Al-Beidh F, Annane D, Arabi Y, Beane A, et al. Effect of Hydrocortisone on Mortality and Organ Support in Patients With Severe COVID-19: The REMAP-CAP COVID-19 Corticosteroid Domain Randomized Clinical Trial. JAMA 2020; 324:1317-1329.

Annane D, Renault A, Brun-Buisson C, Megarbane B, Quenot JP, Siami S, et al. Hydrocortisone plus Fludrocortisone for Adults with Septic Shock. N Engl J Med 2018; 378:809-818.

Annane D, Siami S, Jaber S, Martin C, Elatrous S, Declère AD, et al. Effects of fluid resuscitation with colloids vs crystalloids on mortality in critically ill patients presenting with hypovolemic shock: the CRISTAL randomized trial. JAMA 2013; 310:1809-17.

Annane D, Timsit JF, Megarbane B, Martin C, Misset B, Mourvillier B, et al. Recombinant human activated protein C for adults with septic shock: a randomized controlled trial. Am J Respir Crit Care Med 2013; 187:1091-7.

Arabi YM, Aldawood AS, Haddad SH, Al-Dorzi HM, Tamim HM, Jones G, et al. Permissive Underfeeding or Standard Enteral Feeding in Critically Ill Adults. N Engl J Med 2015; 372:2398-408.

Arabi YM, Aldekhyl S, Al Qahtani S, Al-Dorzi HM, Abdukahil SA, Al Harbi MK et al. Effect of Helmet Noninvasive Ventilation vs Usual Respiratory Support on Mortality Among Patients With Acute Hypoxemic Respiratory Failure Due to COVID-19: The HELMET-COVID Randomized Clinical Trial. JAMA 2022; 328:1063-1072.

Arabi YM, Asiri AY, Assiri AM, Balkhy HH, Al Bshabshe A, Al Jeraisy M, et al. Interferon Beta-1b and Lopinavir-Ritonavir for Middle East Respiratory Syndrome. N Engl J Med 2020; 383:1645-1656.

Arabi YM, Gordon AC, Derde LPG, Nichol AD, Murthy S, Beidh FA, et al. Lopinavir-ritonavir and hydroxychloroquine for critically ill patients with COVID-19: REMAP-CAP randomized controlled trial. Intensive Care Med 2021; 47:867-886.

Asfar P, Meziani F, Hamel JF, Grelon F, Megarbane B, Anguel N, et al. High versus low blood-pressure target in patients with septic shock. N Engl J Med 2014; 370:1583-93.

Azoulay E, Lemiale V, Mokart D, Nseir S, Argaud L, Pène F, et al. Effect of High-Flow Nasal Oxygen vs Standard Oxygen on 28-Day Mortality in Immunocompromised Patients With Acute Respiratory Failure: The HIGH Randomized Clinical Trial. JAMA 2018; 320:2099-2107.

Bagshaw SM, Wald R, Adhikari NKJ, Bellomo R, da Costa BR, Dreyfuss D, et al. Timing of Initiation of Renal-Replacement Therapy in Acute Kidney Injury. N Engl J Med 2020; 383:240-251.

Baksaas-Aasen K, Gall LS, Stensballe J, Juffermans NP, Curry N, Maegele M, et al. Viscoelastic haemostatic assay augmented protocols for major trauma haemorrhage (ITACTIC): a randomized, controlled trial. Intensive Care Med 2021; 47:49-59.

Barbar SD, Clere-Jehl R, Bourredjem A, Hernu R, Montini F, Bruyère R, et al. Timing of Renal-Replacement Therapy in Patients with Acute Kidney Injury and Sepsis. N Engl J Med 2018; 379:1431-1442.

Barrot L, Asfar P, Mauny F, Winiszewski H, Montini F, Badie J, et al. Liberal or Conservative Oxygen Therapy for Acute Respiratory Distress Syndrome. N Engl J Med 2020; 382:999-1008.

Bein T, Weber-Carstens S, Goldmann A, Müller T, Staudinger T, Brederlau J, et al. Lower tidal volume strategy (≈3 ml/kg) combined with extracorporeal CO2 removal versus 'conventional' protective ventilation (6 ml/kg) in severe ARDS: the prospective randomized Xtravent-study. Intensive Care Med 2013; 39:847-56.

Beitler JR, Sarge T, Banner-Goodspeed VM, Gong MN, Cook D, Novack V, et al. Effect of Titrating Positive End-Expiratory Pressure (PEEP) With an Esophageal Pressure-Guided Strategy vs an Empirical High PEEP-Fio2 Strategy on Death and Days Free From Mechanical Ventilation Among Patients With Acute Respiratory Distress Syndrome: A Randomized Clinical Trial. JAMA 2019; 321:846-857.

Belohlavek J, Smalcova J, Rob D, Franek O, Smid O, Pokorna M, et al. Effect of Intra-arrest Transport, Extracorporeal Cardiopulmonary Resuscitation, and Immediate Invasive Assessment and Treatment on Functional Neurologic Outcome in Refractory Out-of-Hospital Cardiac Arrest: A Randomized Clinical Trial. JAMA 2022; 327:737-747.

Bergamin FS, Almeida JP, Landoni G, Galas FRBG, Fukushima JT, Fominskiy E, et al. Liberal Versus Restrictive Transfusion Strategy in Critically Ill Oncologic Patients: The Transfusion Requirements in Critically Ill Oncologic Patients Randomized Controlled Trial. Crit Care Med 2017; 45:766-773.

Bernard GR, Francois B, Mira JP, Vincent JL, Dellinger RP, Russell JA, et al. Evaluating the efficacy and safety of two doses of the polyclonal anti-tumor necrosis factor-α fragment antibody AZD9773 in adult patients with severe sepsis and/or septic shock: randomized, double-blind, placebo-controlled phase IIb study*. Crit Care Med 2014; 42:504-11.

Bloos F, Held J, Kluge S, Simon P, Kogelmann K, de Heer G, et al. (1 → 3)-β-D-Glucan-guided antifungal therapy in adults with sepsis: the CandiSep randomized clinical trial. Intensive Care Med 2022; 48:865-875.

Bloos F, Rüddel H, Thomas-Rüddel D, Schwarzkopf D, Pausch C, Harbarth S, et al. Effect of a multifaceted educational intervention for anti-infectious measures on sepsis mortality: a cluster randomized trial. Intensive Care Med 2017; 43:1602-1612.

Bloos F, Trips E, Nierhaus A, Briegel J, Heyland DK, Jaschinski U, et al. Effect of Sodium Selenite Administration and Procalcitonin-Guided Therapy on Mortality in Patients With Severe Sepsis or Septic Shock: A Randomized Clinical Trial. JAMA Intern Med 2016; 176:1266-76.

Bohé J, Abidi H, Brunot V, Klich A, Klouche K, Sedillot N, et al. Individualised versus conventional glucose control in critically-ill patients: the CONTROLING study-a randomized clinical trial. Intensive Care Med 2021; 47:1271-1283.

Bösel J, Niesen WD, Salih F, Morris NA, Ragland JT, Gough B, et al. Effect of Early vs Standard Approach to Tracheostomy on Functional Outcome at 6 Months Among Patients With Severe Stroke Receiving Mechanical Ventilation: The SETPOINT2 Randomized Clinical Trial. JAMA 2022; 327:1899-1909.

Bouadma L, Mekontso-Dessap A, Burdet C, Merdji H, Poissy J, Dupuis C, et al. High-Dose Dexamethasone and Oxygen Support Strategies in Intensive Care Unit Patients With Severe COVID-19 Acute Hypoxemic Respiratory Failure: The COVIDICUS Randomized Clinical Trial. JAMA Intern Med 2022; 182:906-916.

Bradbury CA, Lawler PR, Stanworth SJ, McVerry BJ, McQuilten Z, Higgins AM, et al. Effect of Antiplatelet Therapy on Survival and Organ Support-Free Days in Critically Ill Patients With COVID-19: A Randomized Clinical Trial. JAMA 2022; 327:1247-1259.

Caironi P, Tognoni G, Masson S, Fumagalli R, Pesenti A, Romero M, et al. Albumin replacement in patients with severe sepsis or septic shock. N Engl J Med 2014; 370:1412-21.

Campos DR, Bueno TBC, Anjos JSGG, Zoppi D, Dantas BG, Gosselink R, et al. Early Neuromuscular Electrical Stimulation in Addition to Early Mobilization Improves Functional Status and Decreases Hospitalization Days of Critically Ill Patients. Crit Care Med 2022; 50:1116-1126.

Carvelli J, Meziani F, Dellamonica J, Cordier PY, Allardet-Servent J, Fraisse M, et al. Avdoralimab (Anti-C5aR1 mAb) Versus Placebo in Patients With Severe COVID-19: Results From a Randomized Controlled Trial (FOR COVID Elimination [FORCE]). Crit Care Med 2022; 50:1788-1798.

Casamento AJ, Serpa Neto A, Young M, Lawrence M, Taplin C, Eastwood GM, et al. A Phase II Cluster-Crossover Randomized Trial of Fentanyl versus Morphine for Analgosedation in Mechanically Ventilated Patients. Am J Respir Crit Care Med 2021; 204:1286-1294.

Cavalcanti AB, Bozza FA, Machado FR, Salluh JI, Campagnucci VP, Vendramim P, et al. Effect of a Quality Improvement Intervention With Daily Round Checklists, Goal Setting, and Clinician Prompting on Mortality of Critically Ill Patients: A Randomized Clinical Trial. JAMA 2016; 315:1480-90.

Cavalcanti AB, Suzumura ÉA, Laranjeira LN, Paisani DM, Damiani LP, Guimarães HP, et al. Effect of Lung Recruitment and Titrated Positive End-Expiratory Pressure (PEEP) vs Low PEEP on Mortality in Patients With Acute Respiratory Distress Syndrome: A Randomized Clinical Trial. JAMA 2017; 318:1335-1345.

Chang P, Liao Y, Guan J, Guo Y, Zhao M, Hu J, et al. Combined Treatment With Hydrocortisone, Vitamin C, and Thiamine for Sepsis and Septic Shock: A Randomized Controlled Trial. Chest 2020; 158:174-182.

Chapman M, Peake SL, Bellomo R, Davies A, Deane A, Horowitz M, et al. Energy-Dense versus Routine Enteral Nutrition in the Critically Ill. N Engl J Med 2018; 379:1823-1834.

Chesnut RM, Temkin N, Carney N, Dikmen S, Rondina C, Videtta W, et al. A trial of intracranial-pressure monitoring in traumatic brain injury. N Engl J Med 2012; 367:2471-81.

Combes A, Bréchot N, Amour J, Cozic N, Lebreton G, Guidon C, et al. Early High-Volume Hemofiltration versus Standard Care for Post-Cardiac Surgery Shock. The HEROICS Study. Am J Respir Crit Care Med 2015; 192:1179-90.

Combes A, Hajage D, Capellier G, Demoule A, Lavoué S, Guervilly C, et al. Extracorporeal Membrane Oxygenation for Severe Acute Respiratory Distress Syndrome. N Engl J Med 2018; 378:1965-1975.

Cooper DJ, McQuilten ZK, Nichol A, Ady B, Aubron C, Bailey M, et al. Age of Red Cells for Transfusion and Outcomes in Critically Ill Adults. N Engl J Med 2017; 377:1858-1867.

Cooper DJ, Nichol AD, Bailey M, Bernard S, Cameron PA, Pili-Floury S, et al. Effect of Early Sustained Prophylactic Hypothermia on Neurologic Outcomes Among Patients With Severe Traumatic Brain Injury: The POLAR Randomized Clinical Trial. JAMA 2018; 320:2211-2220.

CRASH-3 trial collaborators. Effects of tranexamic acid on death, disability, vascular occlusive events and other morbidities in patients with acute traumatic brain injury (CRASH-3): a randomised, placebo-controlled trial. Lancet 2019; 394:1713-1723.

Cuzco C, Castro P, Marín Pérez R, Ruiz García S, Núñez Delgado AI, Romero García M, et al. Impact of a Nurse-Driven Patient Empowerment Intervention on the Reduction in Patients' Anxiety and Depression During ICU Discharge: A Randomized Clinical Trial. Crit Care Med 2022; 50:1757-1767.

Dale CM, Rose L, Carbone S, Pinto R, Smith OM, Burry L, et al. Effect of oral chlorhexidine de-adoption and implementation of an oral care bundle on mortality for mechanically ventilated patients in the intensive care unit (CHORAL): a multi-center stepped wedge cluster-randomized controlled trial. Intensive Care Med 2021; 47:1295-1302.

Dankiewicz J, Cronberg T, Lilja G, Jakobsen JC, Levin H, Ullén S, et al. Hypothermia versus Normothermia after Out-of-Hospital Cardiac Arrest. N Engl J Med 2021; 384:2283-2294.

Daubin C, Valette X, Thiollière F, Mira JP, Hazera P, Annane D, et al. Procalcitonin algorithm to guide initial antibiotic therapy in acute exacerbations of COPD admitted to the ICU: a randomized multicenter study. Intensive Care Med 2018; 44:428-437.

de Almeida JP, Vincent JL, Galas FR, de Almeida EP, Fukushima JT, Osawa EA, et al. Transfusion requirements in surgical oncology patients: a prospective, randomized controlled trial. Anesthesiology 2015; 122:29-38.

Dellinger RP, Bagshaw SM, Antonelli M, Foster DM, Klein DJ, Marshall JC, et al. Effect of Targeted Polymyxin B Hemoperfusion on 28-Day Mortality in Patients With Septic Shock and Elevated Endotoxin Level: The EUPHRATES Randomized Clinical Trial. JAMA 2018; 320:1455-1463.

Dequin PF, Heming N, Meziani F, Plantefève G, Voiriot G, Badié J, et al. Effect of Hydrocortisone on 21-Day Mortality or Respiratory Support Among Critically Ill Patients With COVID-19: A Randomized Clinical Trial. JAMA 2020; 324:1298-1306.

Desch S, Freund A, Akin I, Behnes M, Preusch MR, Zelniker TA, et al. Angiography after Out-of-Hospital Cardiac Arrest without ST-Segment Elevation. N Engl J Med 2021; 385:2544-2553.

Doig GS, Simpson F, Sweetman EA, Finfer SR, Cooper DJ, Heighes PT, et al. Early parenteral nutrition in critically ill patients with short-term relative contraindications to early enteral nutrition: a randomized controlled trial. JAMA 2013; 309:2130-8.

Dulhunty JM, Roberts JA, Davis JS, Webb SA, Bellomo R, Gomersall C, et al. A Multicenter Randomized Trial of Continuous versus Intermittent β-Lactam Infusion in Severe Sepsis. Am J Respir Crit Care Med 2015; 192:1298-305.

Estcourt LJ, Turgeon AF, McQuilten ZK, McVerry BJ, Al-Beidh F, Annane D, et al. Effect of Convalescent Plasma on Organ Support-Free Days in Critically Ill Patients With COVID-19: A Randomized Clinical Trial. JAMA 2021; 326:1690-1702.

Ferguson ND, Cook DJ, Guyatt GH, Mehta S, Hand L, Austin P, et al. High-frequency oscillation in early acute respiratory distress syndrome. N Engl J Med 2013; 368:795-805.

Finfer S, Micallef S, Hammond N, Navarra L, Bellomo R, Billot L, et al. Balanced Multielectrolyte Solution versus Saline in Critically Ill Adults. N Engl J Med 2022; 386:815-826.

Frat JP, Quenot JP, Badie J, Coudroy R, Guitton C, Ehrmann S, et al. Effect of High-Flow Nasal Cannula Oxygen vs Standard Oxygen Therapy on Mortality in Patients With Respiratory Failure Due to COVID-19: The SOHO-COVID Randomized Clinical Trial. JAMA 2022; 328:1212-1222.

Fujii T, Luethi N, Young PJ, Frei DR, Eastwood GM, French CJ, et al. Effect of Vitamin C, Hydrocortisone, and Thiamine vs Hydrocortisone Alone on Time Alive and Free of Vasopressor Support Among Patients With Septic Shock: The VITAMINS Randomized Clinical Trial. JAMA 2020; 323:423-431.

Garbero E, Livigni S, Ferrari F, Finazzi S, Langer M, Malacarne P, et al. High dose coupled plasma filtration and adsorption in septic shock patients. Results of the COMPACT-2: a multicentre, adaptive, randomised clinical trial. Intensive Care Med 2021; 47:1303-1311.

Garrouste-Orgeas M, Flahault C, Vinatier I, Rigaud JP, Thieulot-Rolin N, Mercier E, et al. Effect of an ICU Diary on Posttraumatic Stress Disorder Symptoms Among Patients Receiving Mechanical Ventilation: A Randomized Clinical Trial. JAMA 201; 322:229-239.

Gaudry S, Hajage D, Martin-Lefevre L, Lebbah S, Louis G, Moschietto S, et al. Comparison of two delayed strategies for renal replacement therapy initiation for severe acute kidney injury (AKIKI 2): a multicentre, open-label, randomised, controlled trial. Lancet 2021; 397:1293-1300.

Gaudry S, Hajage D, Schortgen F, Martin-Lefevre L, Pons B, Boulet E, et al. Initiation Strategies for Renal-Replacement Therapy in the Intensive Care Unit. N Engl J Med 2016; 375:122-33.

Ginde AA, Brower RG, Caterino JM, Finck L, Banner-Goodspeed VM, Grissom CK, et al. Early High-Dose Vitamin D3 for Critically Ill, Vitamin D-Deficient Patients. N Engl J Med 2019; 381:2529-2540.

Girard TD, Exline MC, Carson SS, Hough CL, Rock P, Gong MN, et al. Haloperidol and Ziprasidone for Treatment of Delirium in Critical Illness. N Engl J Med 2018; 379:2506-2516.

Girardis M, Busani S, Damiani E, Donati A, Rinaldi L, Marudi A, et al. Effect of Conservative vs Conventional Oxygen Therapy on Mortality Among Patients in an Intensive Care Unit: The Oxygen-ICU Randomized Clinical Trial. JAMA. 2016 Oct 18;316(15):1583-1589.

Goligher EC, Bradbury CA, McVerry BJ, Lawler PR, Berger JS, Gong MN, et al. Therapeutic Anticoagulation with Heparin in Critically Ill Patients with Covid-19. N Engl J Med 2021; 385:777-789.

Gordon AC, Mason AJ, Thirunavukkarasu N, Perkins GD, Cecconi M, Cepkova M, et al. Effect of Early Vasopressin vs Norepinephrine on Kidney Failure in Patients With Septic Shock: The VANISH Randomized Clinical Trial. JAMA 2016; 316:509-18.

Gordon AC, Mouncey PR, Al-Beidh F, Rowan KM, Nichol AD, Arabi YM, et al. Interleukin-6 Receptor Antagonists in Critically Ill Patients with Covid-19. N Engl J Med 2021; 384:1491-1502.

Grieco DL, Menga LS, Cesarano M, Rosà T, Spadaro S, Bitondo MM, et al. Effect of Helmet Noninvasive Ventilation vs High-Flow Nasal Oxygen on Days Free of Respiratory Support in Patients With COVID-19 and Moderate to Severe Hypoxemic Respiratory Failure: The HENIVOT Randomized Clinical Trial. JAMA 2021; 325:1731-1743.

Guérin C, Reignier J, Richard JC, Beuret P, Gacouin A, Boulain T, et al. Prone positioning in severe acute respiratory distress syndrome. N Engl J Med 2013; 368:2159-68.

Guidet B, Leblanc G, Simon T, Woimant M, Quenot JP, Ganansia O, et al. Effect of Systematic Intensive Care Unit Triage on Long-term Mortality Among Critically Ill Elderly Patients in France: A Randomized Clinical Trial. JAMA 2017; 318:1450-1459.

Guntupalli K, Dean N, Morris PE, Bandi V, Margolis B, Rivers E, et al. A phase 2 randomized, double-blind, placebo-controlled study of the safety and efficacy of talactoferrin in patients with severe sepsis. Crit Care Med 2013; 41:706-16.

Hajjar LA, Vincent JL, Barbosa Gomes Galas FR, Rhodes A, Landoni G, Osawa EA, et al. Vasopressin versus Norepinephrine in Patients with Vasoplegic Shock after Cardiac Surgery: The VANCS Randomized Controlled Trial. Anesthesiology 2017; 126:85-93.

Hajjar LA, Zambolim C, Belletti A, de Almeida JP, Gordon AC, Oliveira G, et al. Vasopressin Versus Norepinephrine for the Management of Septic Shock in Cancer Patients: The VANCS II Randomized Clinical Trial. Crit Care Med 2019; 47:1743-1750.

Hanley DF, Lane K, McBee N, Ziai W, Tuhrim S, Lees KR, et al. Thrombolytic removal of intraventricular haemorrhage in treatment of severe stroke: results of the randomised, multicentre, multiregion, placebo-controlled CLEAR III trial. Lancet 2017; 389:603-611.

Harvey SE, Parrott F, Harrison DA, Bear DE, Segaran E, Beale R, et al. rial of the route of early nutritional support in critically ill adults. N Engl J Med 2014; 371:1673-84.

Hassager C, Schmidt H, Møller JE, Grand J, Mølstrøm S, Beske RP, et al. Prevention after Cardiac Arrest. N Engl J Med 2023; 388:888-897.

Hernández G, Ospina-Tascón GA, Damiani LP, Estenssoro E, Dubin A, Hurtado J, et al. Effect of a Resuscitation Strategy Targeting Peripheral Perfusion Status vs Serum Lactate Levels on 28-Day Mortality Among Patients With Septic Shock: The ANDROMEDA-SHOCK Randomized Clinical Trial. JAMA 2019; 321:654-664.

Heyland D, Muscedere J, Wischmeyer PE, Cook D, Jones G, Albert M, et al. Randomized Trial of Glutamine and Antioxidants in Critically Ill Patients. N Engl J Med 2013; 368:1489‐1497.

Heyland DK, Wibbenmeyer L, Pollack JA, Friedman B, Turgeon AF, Eshraghi N, et al. A Randomized Trial of Enteral Glutamine for Treatment of Burn Injuries. N Engl J Med 2022; 387:1001-1010.

Ho KM, Rao S, Honeybul S, Zellweger R, Wibrow B, Lipman J, et al. A Multicenter Trial of Vena Cava Filters in Severely Injured Patients. N Engl J Med 2019; 381:328-337.

Hodgson CL, Bailey M, Bellomo R, Brickell K, Broadley T, Buhr H, et al. Early Active Mobilization during Mechanical Ventilation in the ICU. N Engl J Med 2022; 387:1747-1758.

Hodgson CL, Cooper DJ, Arabi Y, King V, Bersten A, Bihari S, et al. Maximal Recruitment Open Lung Ventilation in Acute Respiratory Distress Syndrome (PHARLAP). A Phase II, Multicenter Randomized Controlled Clinical Trial. Am J Respir Crit Care Med 2019; 200:1363-1372.

Holst LB, Haase N, Wetterslev J, Wernerman J, Guttormsen AB, Karlsson S, et al. Lower versus higher hemoglobin threshold for transfusion in septic shock. N Engl J Med 2014; 371:1381-91.

Hughes CG, Mailloux PT, Devlin JW, Swan JT, Sanders RD, Anzueto A, et al. Dexmedetomidine or Propofol for Sedation in Mechanically Ventilated Adults with Sepsis. N Engl J Med 2021; 384(15):1424-1436.

Ibarra-Estrada MÁ, García-Salas Y, Mireles-Cabodevila E, López-Pulgarín JA, Chávez-Peña Q, García-Salcido R, et al. Use of Airway Pressure Release Ventilation in Patients With Acute Respiratory Failure Due to COVID-19: Results of a Single-Center Randomized Controlled Trial. Crit Care Med 2022; 50:586-594.

INSPIRATION-S Investigators. Atorvastatin versus placebo in patients with covid-19 in intensive care: randomized controlled trial. BMJ 2022; 376:e068407.

Jaber S, Paugam C, Futier E, Lefrant JY, Lasocki S, Lescot T, et al. Sodium bicarbonate therapy for patients with severe metabolic acidaemia in the intensive care unit (BICAR-ICU): a multicentre, open-label, randomised controlled, phase 3 trial. Lancet 2018; 392:31-40.

Jensen JF, Egerod I, Bestle MH, Christensen DF, Elklit A, Hansen RL, et al. A recovery program to improve quality of life, sense of coherence and psychological health in ICU survivors: a multicenter randomized controlled trial, the RAPIT study. Intensive Care Med 2016; 42:1733-1743.

Joannes-Boyau O, Honoré PM, Perez P, Bagshaw SM, Grand H, Canivet JL, et al. High-volume versus standard-volume haemofiltration for septic shock patients with acute kidney injury (IVOIRE study): a multicentre randomized controlled trial. Intensive Care Med 2013; 39:1535-46.

Johansson PI, Søe-Jensen P, Bestle MH, Clausen NE, Kristiansen KT, Lange T, et al. Prostacyclin in Intubated Patients with COVID-19 and Severe Endotheliopathy: A Multicenter, Randomized Clinical Trial. Am J Respir Crit Care Med 2022; 205:324-329.

Jolliet P, Ouanes-Besbes L, Abroug F, Ben Khelil J, Besbes M, Garnero A, et al. A Multicenter Randomized Trial Assessing the Efficacy of Helium/Oxygen in Severe Exacerbations of Chronic Obstructive Pulmonary Disease. Am J Respir Crit Care Med 2017 Apr 1;195(7):871-880.

Jüttler E, Unterberg A, Woitzik J, Bösel J, Amiri H, Sakowitz OW, et al. Hemicraniectomy in older patients with extensive middle-cerebral-artery stroke. N Engl J Med 2014; 370:1091-100.

Kacmarek RM, Villar J, Parrilla D, Alba F, Solano R, Liu S, et al. Neurally adjusted ventilatory assist in acute respiratory failure: a randomized controlled trial. Intensive Care Med 2020; 46:2327-2337.

Kalfon P, Giraudeau B, Ichai C, Guerrini A, Brechot N, Cinotti R, et al. Tight computerized versus conventional glucose control in the ICU: a randomized controlled trial. Intensive Care Med 2014; 40:171-181.

Kawazoe Y, Miyamoto K, Morimoto T, Yamamoto T, Fuke A, Hashimoto A, et al. Effect of Dexmedetomidine on Mortality and Ventilator-Free Days in Patients Requiring Mechanical Ventilation With Sepsis: A Randomized Clinical Trial. JAMA 2017; 317:1321-1328.

Kirkegaard H, Søreide E, de Haas I, Pettilä V, Taccone FS, Arus U, et al. Targeted Temperature Management for 48 vs 24 Hours and Neurologic Outcome After Out-of-Hospital Cardiac Arrest: A Randomized Clinical Trial. JAMA 2017; 318:341-350.

Kjaergaard J, Møller JE, Schmidt H, Grand J, Mølstrøm S, Borregaard B, et al. Blood-Pressure Targets in Comatose Survivors of Cardiac Arrest. N Engl J Med 2022; 387:1456-1466.

Krag M, Marker S, Perner A, Wetterslev J, Wise MP, Schefold JC, et al. Pantoprazole in Patients at Risk for Gastrointestinal Bleeding in the ICU. N Engl J Med 2018; 379:2199-2208.

Lacroix J, Hébert PC, Fergusson DA, Tinmouth A, Cook DJ, Marshall JC, et al. Age of transfused blood in critically ill adults. N Engl J Med 2015; 372:1410-8.

Lamontagne F, Masse MH, Menard J, Sprague S, Pinto R, Heyland DK, et al. Intravenous Vitamin C in Adults with Sepsis in the Intensive Care Unit. N Engl J Med 2022; 386:2387-2398.

Lamontagne F, Richards-Belle A, Thomas K, Harrison DA, Sadique MZ, Grieve RD, et al. Effect of Reduced Exposure to Vasopressors on 90-Day Mortality in Older Critically Ill Patients With Vasodilatory Hypotension: A Randomized Clinical Trial. JAMA 2020; 323:938-949.

Landoni G, Lomivorotov VV, Alvaro G, Lobreglio R, Pisano A, Guarracino F, et al. Levosimendan for Hemodynamic Support after Cardiac Surgery. N Engl J Med 2017; 376:2021-2031.

Lascarrou JB, Merdji H, Le Gouge A, Colin G, Grillet G, Girardie P, et al. Targeted Temperature Management for Cardiac Arrest with Nonshockable Rhythm. N Engl J Med 2019; 381:2327-2337.

Laterre PF, Berry SM, Blemings A, Carlsen JE, François B, Graves T, et al. Effect of Selepressin vs Placebo on Ventilator- and Vasopressor-Free Days in Patients With Septic Shock: The SEPSIS-ACT Randomized Clinical Trial. JAMA 2019; 322:1476-1485.

Le May M, Osborne C, Russo J, So D, Chong AY, Dick A, et al. Effect of Moderate vs Mild Therapeutic Hypothermia on Mortality and Neurologic Outcomes in Comatose Survivors of Out-of-Hospital Cardiac Arrest: The CAPITAL CHILL Randomized Clinical Trial. JAMA 2021; 326:1494-1503.

Legriel S, Lemiale V, Schenck M, Chelly J, Laurent V, Daviaud F, et al. Hypothermia for Neuroprotection in Convulsive Status Epilepticus. N Engl J Med 2016;375:2457-2467.

Lemiale V, Mokart D, Resche-Rigon M, Pène F, Mayaux J, Faucher E, et al. Effect of Noninvasive Ventilation vs Oxygen Therapy on Mortality Among Immunocompromised Patients With Acute Respiratory Failure: A Randomized Clinical Trial. JAMA 2015;314:1711-9.

Lemkes JS, Janssens GN, van der Hoeven NW, Jewbali LSD, Dubois EA, Meuwissen M, et al. Coronary Angiography after Cardiac Arrest without ST-Segment Elevation. N Engl J Med 2019; 380:1397-1407.

Levy B, Girerd N, Amour J, Besnier E, Nesseler N, Helms J, et al. Effect of Moderate Hypothermia vs Normothermia on 30-Day Mortality in Patients With Cardiogenic Shock Receiving Venoarterial Extracorporeal Membrane Oxygenation: A Randomized Clinical Trial. JAMA 2022; 327:442-453.

Li L, Zhang W, Hu Y, Tong X, Zheng S, Yang J, et al. Effect of Convalescent Plasma Therapy on Time to Clinical Improvement in Patients With Severe and Life-threatening COVID-19: A Randomized Clinical Trial. JAMA 2020; 324:460-470.

Litton E, Anstey M, Broadhurst D, Chapman A, Currie A, Ferrier J, et al. Early and sustained Lactobacillus plantarum probiotic therapy in critical illness: the randomised, placebo-controlled, restoration of gut microflora in critical illness trial (ROCIT). Intensive Care Med 2021; 47:307-315.

Liu ZM, Chen J, Kou Q, Lin Q, Huang X, Tang Z, et al. Terlipressin versus norepinephrine as infusion in patients with septic shock: a multicentre, randomised, double-blinded trial. Intensive Care Med 2018; 44:1816-1825.

Luyt CE, Forel JM, Hajage D, Jaber S, Cayot-Constantin S, Rimmelé T, et al. Acyclovir for Mechanically Ventilated Patients With Herpes Simplex Virus Oropharyngeal Reactivation: A Randomized Clinical Trial. JAMA Intern Med 2020; 180:263-272.

Mackle D, Bellomo R, Bailey M, Beasley R, Deane A, Eastwood G, et al. Conservative Oxygen Therapy during Mechanical Ventilation in the ICU. N Engl J Med 2020; 382:989-998.

Madsen MB, Hjortrup PB, Hansen MB, Lange T, Norrby-Teglund A, Hyldegaard O, et al. Immunoglobulin G for patients with necrotising soft tissue infection (INSTINCT): a randomised, blinded, placebo-controlled trial. Intensive Care Med 2017; 43:1585-1593.

Maitre B, Djibre M, Katsahian S, Habibi A, Stankovic Stojanovic K, Khellaf M, et al. Inhaled nitric oxide for acute chest syndrome in adult sickle cell patients: a randomized controlled study. Intensive Care Med 2015; 41:2121-9.

Mathew R, Di Santo P, Jung RG, Marbach JA, Hutson J, Simard T, et al. Milrinone as Compared with Dobutamine in the Treatment of Cardiogenic Shock. N Engl J Med 2021; 385:516-525.

Mazer CD, Whitlock RP, Fergusson DA, Hall J, Belley-Cote E, Connolly K, et al. Restrictive or Liberal Red-Cell Transfusion for Cardiac Surgery. N Engl J Med 2017; 377:2133-2144.

McAuley DF, Laffey JG, O'Kane CM, Perkins GD, Mullan B, Trinder TJ, et al. Simvastatin in the acute respiratory distress syndrome. N Engl J Med 2014; 371:1695-703.

McNamee JJ, Gillies MA, Barrett NA, Perkins GD, Tunnicliffe W, Young D, et al. Effect of Lower Tidal Volume Ventilation Facilitated by Extracorporeal Carbon Dioxide Removal vs Standard Care Ventilation on 90-Day Mortality in Patients With Acute Hypoxemic Respiratory Failure: The REST Randomized Clinical Trial. JAMA. 2021 Sep 21;326(11):1013-1023.

Meduri GU, Shih MC, Bridges L, Martin TJ, El-Solh A, Seam N, et al. Low-dose methylprednisolone treatment in critically ill patients with severe community-acquired pneumonia. Intensive Care Med 2022; 48:1009-1023.

Mehta RH, Leimberger JD, van Diepen S, Meza J, Wang A, Jankowich R, et al. Levosimendan in Patients with Left Ventricular Dysfunction Undergoing Cardiac Surgery. N Engl J Med 2017; 376:2032-2042.

Meyhoff TS, Hjortrup PB, Wetterslev J, Sivapalan P, Laake JH, Cronhjort M et al. Restriction of Intravenous Fluid in ICU Patients with Septic Shock. N Engl J Med 2022; 386:2459-2470.

Moss M, Huang DT, Brower RG, Ferguson ND, Ginde AA, Gong MN, et al. Early Neuromuscular Blockade in the Acute Respiratory Distress Syndrome. N Engl J Med 2019; 380:1997-2008.

Mouncey PR, Osborn TM, Power GS, Harrison DA, Sadique MZ, Grieve RD, et al. Trial of early, goal-directed resuscitation for septic shock. N Engl J Med 2015; 372:1301-11.

Mourvillier B, Tubach F, van de Beek D, Garot D, Pichon N, Georges H, et al. Induced hypothermia in severe bacterial meningitis: a randomized clinical trial. JAMA 2013; 310:2174-83.

Munch MW, Myatra SN, Vijayaraghavan BKT, Saseedharan S, Benfield T, Wahlin RR, et al. Effect of 12 mg vs 6 mg of Dexamethasone on the Number of Days Alive Without Life Support in Adults With COVID-19 and Severe Hypoxemia: The COVID STEROID 2 Randomized Trial. JAMA 2021; 326:1807-1817.

Myburgh JA, Finfer S, Bellomo R, Billot L, Cass A, Gattas D, et al. Hydroxyethyl starch or saline for fluid resuscitation in intensive care. N Engl J Med 2012; 367:1901-11.

Myburgh JA, Seppelt IM, Goodman F, Billot L, Correa M, Davis JS, et al. Effect of Selective Decontamination of the Digestive Tract on Hospital Mortality in Critically Ill Patients Receiving Mechanical Ventilation: A Randomized Clinical Trial. JAMA 2022; 328:1911-1921.

Nielsen N, Wetterslev J, Cronberg T, Erlinge D, Gasche Y, Hassager C, et al. Targeted temperature management at 33°C versus 36°C after cardiac arrest. N Engl J Med 2013; 369:2197-206.

Olsen HT, Nedergaard HK, Strøm T, Oxlund J, Wian KA, Ytrebø LM, et al. Nonsedation or Light Sedation in Critically Ill, Mechanically Ventilated Patients. N Engl J Med 2020; 382:1103-1111.

Opal SM, Laterre PF, Francois B, LaRosa SP, Angus DC, Mira JP, et al. Effect of eritoran, an antagonist of MD2-TLR4, on mortality in patients with severe sepsis: the ACCESS randomized trial. JAMA 2013; 309:1154-62.

Osawa EA, Rhodes A, Landoni G, Galas FR, Fukushima JT, Park CH, et al. Effect of Perioperative Goal-Directed Hemodynamic Resuscitation Therapy on Outcomes Following Cardiac Surgery: A Randomized Clinical Trial and Systematic Review. Crit Care Med 2016; 44:724-33.

Ospina-Tascón GA, Calderón-Tapia LE, García AF, Zarama V, Gómez-Álvarez F, Álvarez-Saa T, et al. Effect of High-Flow Oxygen Therapy vs Conventional Oxygen Therapy on Invasive Mechanical Ventilation and Clinical Recovery in Patients With Severe COVID-19: A Randomized Clinical Trial. JAMA 2021; 326:2161-2171.

Papazian L, Roch A, Charles PE, Penot-Ragon C, Perrin G, Roulier P, et al. Effect of statin therapy on mortality in patients with ventilator-associated pneumonia: a randomized clinical trial. JAMA 2013; 310:1692-700.

Pappalardo F, Crivellari M, Di Prima AL, Agracheva N, Celinska-Spodar M, Lembo R, et al. Protein C zymogen in severe sepsis: a double-blinded, placebo-controlled, randomized study. Intensive Care Med 2016; 42:1706-1714.

Payen DM, Guilhot J, Launey Y, Lukaszewicz AC, Kaaki M, Veber B, et al. Early use of polymyxin B hemoperfusion in patients with septic shock due to peritonitis: a multicenter randomized control trial. Intensive Care Med 2015; 41:975-84.

Peake SL, Delaney A, Bailey M, Bellomo R, Cameron PA, Cooper DJ, et al. Goal-directed resuscitation for patients with early septic shock. N Engl J Med. 2014; 371:1496-506.

Perkins GD, Ji C, Connolly BA, Couper K, Lall R, Baillie JK, et al. Effect of Noninvasive Respiratory Strategies on Intubation or Mortality Among Patients With Acute Hypoxemic Respiratory Failure and COVID-19: The RECOVERY-RS Randomized Clinical Trial. JAMA 2022; 327:546-558.

Pesonen E, Vlasov H, Suojaranta R, Hiippala S, Schramko A, Wilkman E, et al. Effect of 4% Albumin Solution vs Ringer Acetate on Major Adverse Events in Patients Undergoing Cardiac Surgery With Cardiopulmonary Bypass: A Randomized Clinical Trial. JAMA 2022; 328:251-258.

Quenot JP, Binquet C, Vinsonneau C, Barbar SD, Vinault S, Deckert V, et al. Very high volume hemofiltration with the Cascade system in septic shock patients. Intensive Care Med 2015; 41:2111-20.

Ranieri VM, Pettilä V, Karvonen MK, Jalkanen J, Nightingale P, Brealey D, et al. Effect of Intravenous Interferon β-1a on Death and Days Free From Mechanical Ventilation Among Patients With Moderate to Severe Acute Respiratory Distress Syndrome: A Randomized Clinical Trial. JAMA 2020; 323:725-733.

Ranucci M, Castelvecchio S, Biondi A, de Vincentiis C, Ballotta A, Varrica A, et al. A randomized controlled trial of preoperative intra-aortic balloon pump in coronary patients with poor left ventricular function undergoing coronary artery bypass surgery*. Crit Care Med 2013; 41:2476-83.

Reade MC, Eastwood GM, Bellomo R, Bailey M, Bersten A, Cheung B et al. Effect of Dexmedetomidine Added to Standard Care on Ventilator-Free Time in Patients With Agitated Delirium: A Randomized Clinical Trial. JAMA 2016; 315:1460-8.

Reignier J, Boisramé-Helms J, Brisard L, Lascarrou JB, Ait Hssain A, Anguel N, et al. Enteral versus parenteral early nutrition in ventilated adults with shock: a randomised, controlled, multicentre, open-label, parallel-group study (NUTRIREA-2). Lancet 2018; 391:133-143.

Rein L, Calero K, Shah R, Ojielo C, Hudock KM, Lodhi S, et al. Randomized Phase 3 Trial of Ruxolitinib for COVID-19-Associated Acute Respiratory Distress Syndrome. Crit Care Med 2022; 50:1701-1713.

Robertson CS, Hannay HJ, Yamal JM, Gopinath S, Goodman JC, Tilley BC et al. Effect of erythropoietin and transfusion threshold on neurological recovery after traumatic brain injury: a randomized clinical trial. JAMA 2014; 312:36-47

Rocha Ferreira GS, de Almeida JP, Landoni G, Vincent JL, Fominskiy E, Gomes Galas FRB, et al. Effect of a Perioperative Intra-Aortic Balloon Pump in High-Risk Cardiac Surgery Patients: A Randomized Clinical Trial. Crit Care Med 2018; 46:e742-e750.

Roquilly A, Moyer JD, Huet O, Lasocki S, Cohen B, Dahyot-Fizelier C, et al. Effect of Continuous Infusion of Hypertonic Saline vs Standard Care on 6-Month Neurological Outcomes in Patients With Traumatic Brain Injury: The COBI Randomized Clinical Trial. JAMA 2021; 325:2056-2066.

Rosas IO, Bräu N, Waters M, Go RC, Hunter BD, Bhagani S, et al. Tocilizumab in Hospitalized Patients with Severe Covid-19 Pneumonia. N Engl J Med 2021; 384:1503-1516.

Ruijter BJ, Keijzer HM, Tjepkema-Cloostermans MC, Blans MJ, Beishuizen A, Tromp SC, et al. Treating Rhythmic and Periodic EEG Patterns in Comatose Survivors of Cardiac Arrest. N Engl J Med 2022; 386:724-734.

Russell DW, Casey JD, Gibbs KW, Ghamande S, Dargin JM, Vonderhaar DJ, et al. Effect of Fluid Bolus Administration on Cardiovascular Collapse Among Critically Ill Patients Undergoing Tracheal Intubation: A Randomized Clinical Trial. JAMA 2022; 328:270-279.

Sadeghipour P, Talasaz AH, Rashidi F, Sharif-Kashani B, Beigmohammadi MT, Farrokhpour M, et al. Effect of Intermediate-Dose vs Standard-Dose Prophylactic Anticoagulation on Thrombotic Events, Extracorporeal Membrane Oxygenation Treatment, or Mortality Among Patients With COVID-19 Admitted to the Intensive Care Unit: The INSPIRATION Randomized Clinical Trial. JAMA 2021; 325:1620-1630.

Schjørring OL, Klitgaard TL, Perner A, Wetterslev J, Lange T, Siegemund M, et al. Lower or Higher Oxygenation Targets for Acute Hypoxemic Respiratory Failure. N Engl J Med 2021; 384:1301-1311.

Schmidt H, Kjaergaard J, Hassager C, Mølstrøm S, Grand J, Borregaard B, et al. Oxygen Targets in Comatose Survivors of Cardiac Arrest. N Engl J Med 2022; 387:1467-1476.

Schujmann DS, Teixeira Gomes T, Lunardi AC, Zoccoler Lamano M, Fragoso A, Pimentel M, et al. Impact of a Progressive Mobility Program on the Functional Status, Respiratory, and Muscular Systems of ICU Patients: A Randomized and Controlled Trial. Crit Care Med 2020; 48:491-497.

Sehgal IS, Agarwal R, Aggarwal AN, Jindal SK. A randomized trial of Mycobacterium w in severe sepsis. J Crit Care 2015; 30:85-9.

Sehgal IS, Basumatary NM, Dhooria S, Prasad KT, Muthu V, Aggarwal AN, et al. A Randomized Trial of Mycobacterium w in Severe Presumed Gram-Negative Sepsis. Chest 2021; 160:1282-1291.

Semler MW, Casey JD, Lloyd BD, Hastings PG, Hays MA, Stollings JL, et al. Oxygen-Saturation Targets for Critically Ill Adults Receiving Mechanical Ventilation. N Engl J Med 2022; 387:1759-1769.

Semler MW, Self WH, Wanderer JP, Ehrenfeld JM, Wang L, Byrne DW, et al. Balanced Crystalloids versus Saline in Critically Ill Adults. N Engl J Med 2018; 378:829-839.

Sevransky JE, Rothman RE, Hager DN, Bernard GR, Brown SM, Buchman TG, et al. Effect of Vitamin C, Thiamine, and Hydrocortisone on Ventilator- and Vasopressor-Free Days in Patients With Sepsis: The VICTAS Randomized Clinical Trial. JAMA 2021;325:742-750.

Shehabi Y, Howe BD, Bellomo R, Arabi YM, Bailey M, Bass FE, et al. Early Sedation with Dexmedetomidine in Critically Ill Patients. N Engl J Med 2019; 380:2506-2517.

Simonis FD, Serpa Neto A, Binnekade JM, Braber A, Bruin KCM, Determann RM, et al. Effect of a Low vs Intermediate Tidal Volume Strategy on Ventilator-Free Days in Intensive Care Unit Patients Without ARDS: A Randomized Clinical Trial. JAMA. 2018; 320:1872-1880.

Thiele H, Akin I, Sandri M, Fuernau G, de Waha S, Meyer-Saraei R, et al. PCI Strategies in Patients with Acute Myocardial Infarction and Cardiogenic Shock. N Engl J Med 2017; 377:2419-2432.

Thiele H, Zeymer U, Neumann FJ, Ferenc M, Olbrich HG, Hausleiter J, et al. Intraaortic balloon support for myocardial infarction with cardiogenic shock. N Engl J Med 2012; 367:1287-96.

Thille AW, Gacouin A, Coudroy R, Ehrmann S, Quenot JP, Nay MA, et al. Spontaneous-Breathing Trials with Pressure-Support Ventilation or a T-Piece. N Engl J Med 2022; 387:1843-1854.

Timsit JF, Azoulay E, Schwebel C, Charles PE, Cornet M, Souweine B, et al. Empirical Micafungin Treatment and Survival Without Invasive Fungal Infection in Adults With ICU-Acquired Sepsis, Candida Colonization, and Multiple Organ Failure: The EMPIRICUS Randomized Clinical Trial. JAMA 2016; 316:1555-1564.

Tomazini BM, Maia IS, Cavalcanti AB, Berwanger O, Rosa RG, Veiga VC et al. Effect of Dexamethasone on Days Alive and Ventilator-Free in Patients With Moderate or Severe Acute Respiratory Distress Syndrome and COVID-19: The CoDEX Randomized Clinical Trial. JAMA 2020; 324:1307-1316.

Torres A, Sibila O, Ferrer M, Polverino E, Menendez R, Mensa J, et al. Effect of corticosteroids on treatment failure among hospitalized patients with severe community-acquired pneumonia and high inflammatory response: a randomized clinical trial. JAMA 2015; 313:677-86.

Truwit JD, Bernard GR, Steingrub J, Matthay MA, Liu KD, Albertson TE, et al. Rosuvastatin for sepsis-associated acute respiratory distress syndrome. N Engl J Med 2014; 370:2191-200.

van den Boogaard M, Slooter AJC, Brüggemann RJM, Schoonhoven L, Beishuizen A, Vermeijden JW, et al. Effect of Haloperidol on Survival Among Critically Ill Adults With a High Risk of Delirium: The REDUCE Randomized Clinical Trial. JAMA 2018; 319:680-690.

Venkatesh B, Finfer S, Cohen J, Rajbhandari D, Arabi Y, Bellomo R, et al. Adjunctive Glucocorticoid Therapy in Patients with Septic Shock. N Engl J Med 2018; 378:797-808.

Vignon P, Dequin PF, Renault A, Mathonnet A, Paleiron N, Imbert A, et al. Intermittent pneumatic compression to prevent venous thromboembolism in patients with high risk of bleeding hospitalized in intensive care units: the CIREA1 randomized trial. Intensive Care Med 2013; 39:872-80.

Vincent JL, Francois B, Zabolotskikh I, Daga MK, Lascarrou JB, Kirov MY, et al. Effect of a Recombinant Human Soluble Thrombomodulin on Mortality in Patients With Sepsis-Associated Coagulopathy: The SCARLET Randomized Clinical Trial. JAMA 2019; 321:1993-2002.

Vincent JL, Marshall JC, Dellinger RP, Simonson SG, Guntupalli K, Levy MM, et al. Talactoferrin in Severe Sepsis: Results From the Phase II/III Oral tAlactoferrin in Severe sepsIS Trial. Crit Care Med 2015; 43:1832-8.

Vincent JL, Privalle CT, Singer M, Lorente JA, Boehm E, Meier-Hellmann A, et al. Multicenter, randomized, placebo-controlled phase III study of pyridoxalated hemoglobin polyoxyethylene in distributive shock (PHOENIX). Crit Care Med 2015; 43:57-64.

Vincent JL, Ramesh MK, Ernest D, LaRosa SP, Pachl J, Aikawa N, et al. A Randomized, Double-Blind, Placebo-Controlled, Phase 2b Study to Evaluate the Safety and Efficacy of Recombinant Human Soluble Thrombomodulin, ART-123, in Patients With Sepsis and Suspected Disseminated Intravascular Coagulation. Crit Care Med 2013; 41:2069-79.

Wacker DA, Burton SL, Berger JP, Hegg AJ, Heisdorffer J, Wang Q, et al. Evaluating Vitamin C in Septic Shock: A Randomized Controlled Trial of Vitamin C Monotherapy. Crit Care Med 2022; 50:e458-e467.

Wade DM, Mouncey PR, Richards-Belle A, Wulff J, Harrison DA, Sadique MZ, et al. Effect of a Nurse-Led Preventive Psychological Intervention on Symptoms of Posttraumatic Stress Disorder Among Critically Ill Patients: A Randomized Clinical Trial. JAMA 2019; 321:665-675.

Welte T, Dellinger RP, Ebelt H, Ferrer M, Opal SM, Singer M, et al. Efficacy and safety of trimodulin, a novel polyclonal antibody preparation, in patients with severe community-acquired pneumonia: a randomized, placebo-controlled, double-blind, multicenter, phase II trial (CIGMA study). Intensive Care Med 2018; 44:438-448.

Yannopoulos D, Bartos J, Raveendran G, Walser E, Connett J, Murray TA, et al. Advanced reperfusion strategies for patients with out-of-hospital cardiac arrest and refractory ventricular fibrillation (ARREST): a phase 2, single centre, open-label, randomised controlled trial. Lancet 2020; 396:1807-1816.

Yealy DM, Kellum JA, Huang DT, Barnato AE, Weissfeld LA, Pike F, et al. A randomized trial of protocol-based care for early septic shock. N Engl J Med 2014; 370:1683-93.

Young D, Harrison DA, Cuthbertson BH, Rowan K; TracMan Collaborators. Effect of early vs late tracheostomy placement on survival in patients receiving mechanical ventilation: the TracMan randomized trial. JAMA 2013; 309:2121-9.

Young D, Lamb SE, Shah S, MacKenzie I, Tunnicliffe W, Lall R, et al. High-frequency oscillation for acute respiratory distress syndrome. N Engl J Med 2013; 368:806-13.

Young P, Saxena M, Bellomo R, Freebairn R, Hammond N, van Haren F, et al. Acetaminophen for Fever in Critically Ill Patients with Suspected Infection. N Engl J Med 2015; 373:2215-24.

Youssef JG, Lavin P, Schoenfeld DA, Lee RA, Lenhardt R, Park DJ, et al. The Use of IV Vasoactive Intestinal Peptide (Aviptadil) in Patients With Critical COVID-19 Respiratory Failure: Results of a 60-Day Randomized Controlled Trial. Crit Care Med 2022; 50:1545-1554.

Zampieri FG, Machado FR, Biondi RS, Freitas FGR, Veiga VC, Figueiredo RC, et al. Effect of Intravenous Fluid Treatment With a Balanced Solution vs 0.9% Saline Solution on Mortality in Critically Ill Patients: The BaSICS Randomized Clinical Trial. JAMA 2021; 326:1–12.

Zampieri FG, Machado FR, Biondi RS, Freitas FGR, Veiga VC, Figueiredo RC, et al. Effect of Slower vs Faster Intravenous Fluid Bolus Rates on Mortality in Critically Ill Patients: The BaSICS Randomized Clinical Trial. JAMA 2021; 326:830-838.

Zarbock A, Kellum JA, Schmidt C, Van Aken H, Wempe C, Pavenstädt H, et al. Effect of Early vs Delayed Initiation of Renal Replacement Therapy on Mortality in Critically Ill Patients With Acute Kidney Injury: The ELAIN Randomized Clinical Trial. JAMA 2016; 315:2190-9.

Zarbock A, Küllmar M, Kindgen-Milles D, Wempe C, Gerss J, Brandenburger T, et al. Effect of Regional Citrate Anticoagulation vs Systemic Heparin Anticoagulation During Continuous Kidney Replacement Therapy on Dialysis Filter Life Span and Mortality Among Critically Ill Patients With Acute Kidney Injury: A Randomized Clinical Trial. JAMA 2020; 324:1629-1639.

Zhang Q, Li C, Shao F, Zhao L, Wang M, Fang Y. Efficacy and Safety of Combination Therapy of Shenfu Injection and Postresuscitation Bundle in Patients With Return of Spontaneous Circulation After In-Hospital Cardiac Arrest: A Randomized, Assessor-Blinded, Controlled Trial. Crit Care Med 2017; 45:1587-1595.

Zhang Z, Ni H, Qian Z. Effectiveness of treatment based on PiCCO parameters in critically ill patients with septic shock and/or acute respiratory distress syndrome: a randomized controlled trial. Intensive Care Med 2015; 41:444-51.

Zhou Y, Jin X, Lv Y, Wang P, Yang Y, Liang G, et al. Early application of airway pressure release ventilation may reduce the duration of mechanical ventilation in acute respiratory distress syndrome. Intensive Care Med 2017; 43:1648-1659.

**Table S1: Inclusion and Exclusion Criteria**

| **Inclusion** | **Exclusion** |
| --- | --- |
| Randomised Controlled Trial published 1^st^ August 2012 until 31^st^ December 2022 | Any non-randomised controlled trial (this includes pilot studies, secondary reanalyses, studies in abstract form only, nested sub-studies, sub-analysis, post-hoc analysis, or longitudinal follow up studies of RCTs) |
| Adult patients (≥ 18 years old) | Paediatric (< 18 years old) or animal studies |
| Critical Care Population - at least 50% of the trial population was admitted to a critical care unit or receiving a critical care treatment (such as NIV, IMV, Vasoactive medications, CRRT) | Trials involving non-critical care populations |
| A patient important primary outcome (either as primary or as at least one component of a composite outcome) | Any trial that does not include a patient important outcome as a primary outcome or as a component of a composite outcome |
| Journals: NEJM, JAMA, Lancet, BMJ, Critical Care Medicine, Intensive Care Medicine, Chest, American Journal of Respiratory and Critical Care Medicine, Anesthesia and Analgesia, Anesthesiology, Annals of Internal Medicine, JAMA Internal Medicine, British Journal of Anaesthesia (BJA), Canadian Medical Association Journal, Journal of Critical Care, Journal of Trauma and Acute Care Surgery |  |
| English language only |  |

**Table S2 showing all COMs used within the systematic review**

| **Categorical COMs - all include mortality and the following** | **Continuous COMs** |
| --- | --- |
| CPC 3 or 4 | Ventilator Free Days |
| Requirement for Respiratory Support | Ventilator Free Hours |
| RRT requirement / Perioperative MI / Mechanical Cardiac Assist Device | Death and Days Free from Mechanical Ventilation |
| Symptomatic PE | Time to Discharge Alive from the Hospital |
| Need for endotracheal intubation / Worsening oxygenation / Intensification of Therapy | Ventilator and Vasopressor Free Days |
| ECMO / Venous thromboembolism / Arterial thromboembolism | Days Alive and Free from ICU |
| Symptomatic DVT or PE / Asymptomatic DVT | Catecholamine Free Days |
| Prolonged Mechanical Ventilation > 48 hrs / AKI / Stroke / Mediastinitis / Operative Mortality / Re-operation / | SF-36 Score and Mortality* |
| CV Complications / ARDS / AKI needing RRT / Mechanical Ventilation / Septic Shock | Kidney Failure Free Days |
| Low Cardiac Output / Stroke / MI / Re-operation / RRT | Days Alive without Delirium or Coma |
| Mechanical Ventilation > 48 hours / Sternal Wound Infection / Reoperation / AKI / Stroke | Time Alive and Free of Vasopressors |
| RRT / Creatinine > 200% baseline | Organ Support Free Days ** |
| Mechanical Ventilation > 24 hours / Wound Infection / Re-operation / Cardiogenic Shock / Stroke / AKI | Days Alive without Life Supports *** |
| Massive Transfusion | Measures of survival, duration and level of impaired consciousness, functional status, orientation and neuropsychological status |
| Cardiac Arrest / Cardiac Transplant / Mechanical Circulatory Support / MI / TIA or Stroke / RRT |  |
| Disability Rating Score > 5 |  |
| Need for Intubation |  |
| Receipt of vasopressors / Receipt of RRT / Receipt of Mechanical Ventilation |  |
| SBP < 65 mmHg / Cardiac Arrest / New or increased receipt of vasopressors |  |
| MI / New onset heart failure needing re-hospitalisation or intervention / Resternotomy / Stroke / Major Arrythmia / Major Bleeding / AKI / Infection |  |
| Freedom from Fungal Infection |  |

*CPC = cerebral performance category, MI = myocardial infarction, RRT = renal replacement therapy, PE = pulmonary embolism, DVT = deep vein thrombosis, ECMO = extra-corporeal membrane oxygenation, AKI = acute kidney injury, SBP = systolic blood pressure, CV = cardiovascular*

* SF-36 score does not include mortality however the authors scored those that died 0

**Organ Support defined as days alive and free of ICU based respiratory or cardiovascular support

***Life Support included mechanical ventilation, circulatory support and kidney replacement therapy

NB: The exact definitions and time frames used are not reported here

**All Populations Included**


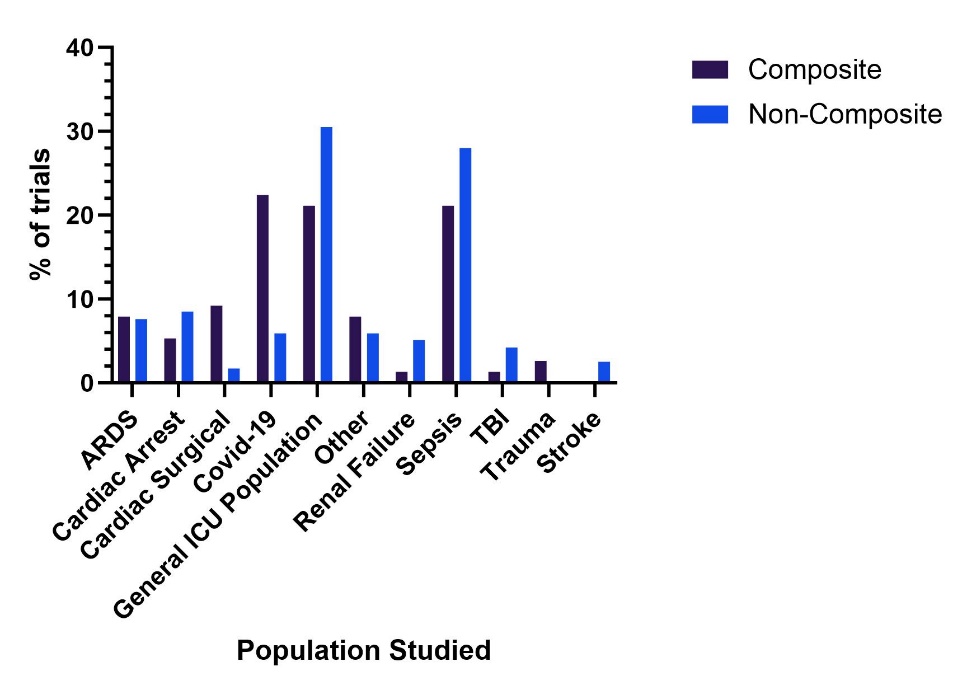
Figure S1 showing populations included

**All Interventions Studied**

Figure S2 showing interventions studied


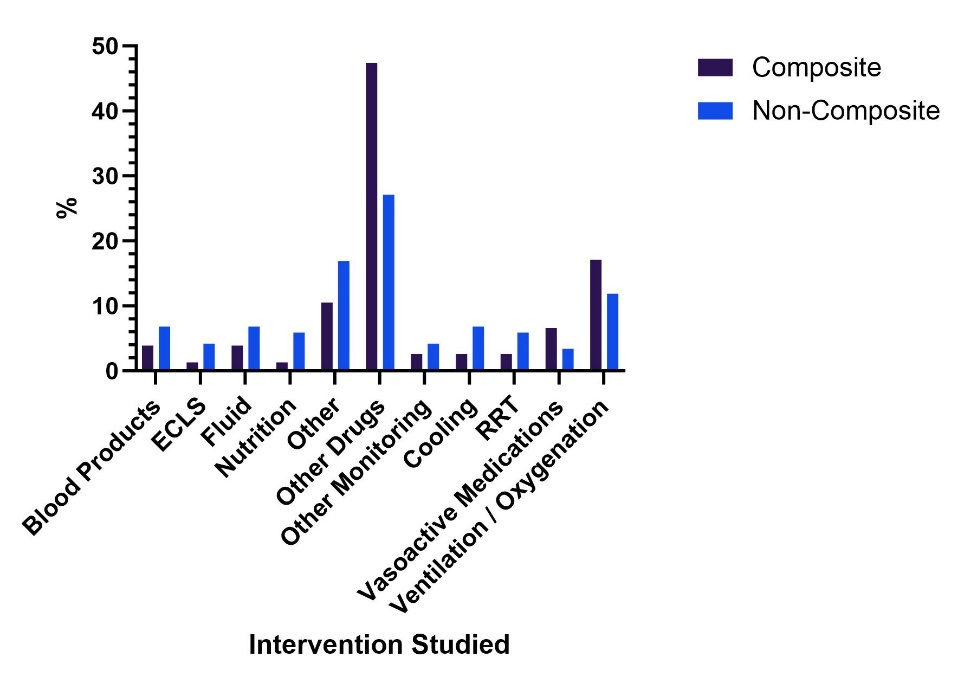


**Risk of Bias Summary**

Figure S3 showing Risk of Bias


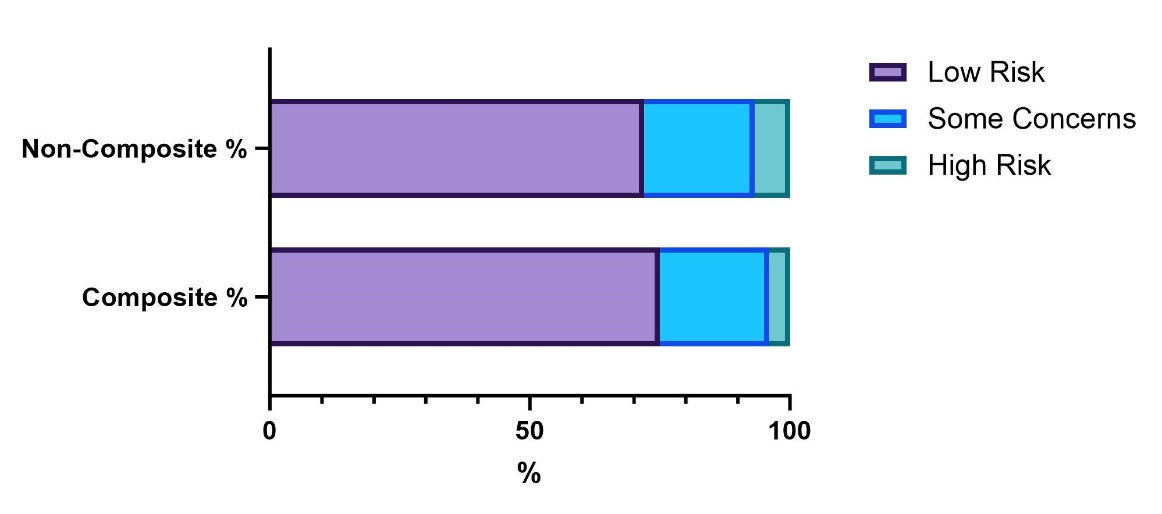


**Study Power**

Figure S4 showing violin plot depicting reported study power for composite and non-composite trials

**
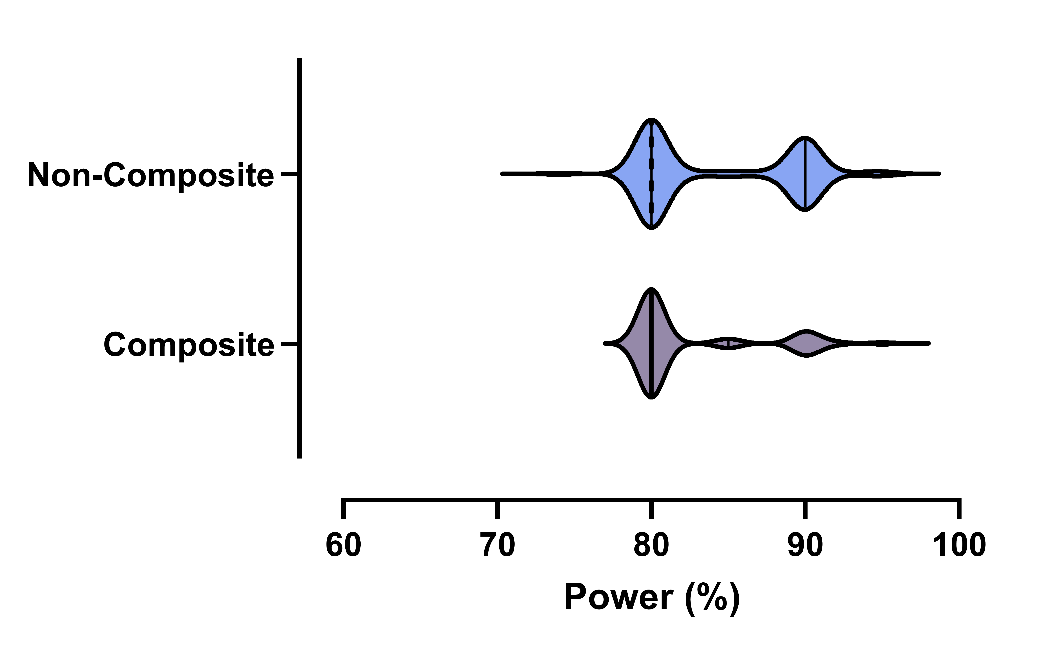
**

**Effect Sizes**

Figure S5 showing the predicted and achieved effect sizes for composite and non-composite studies

**
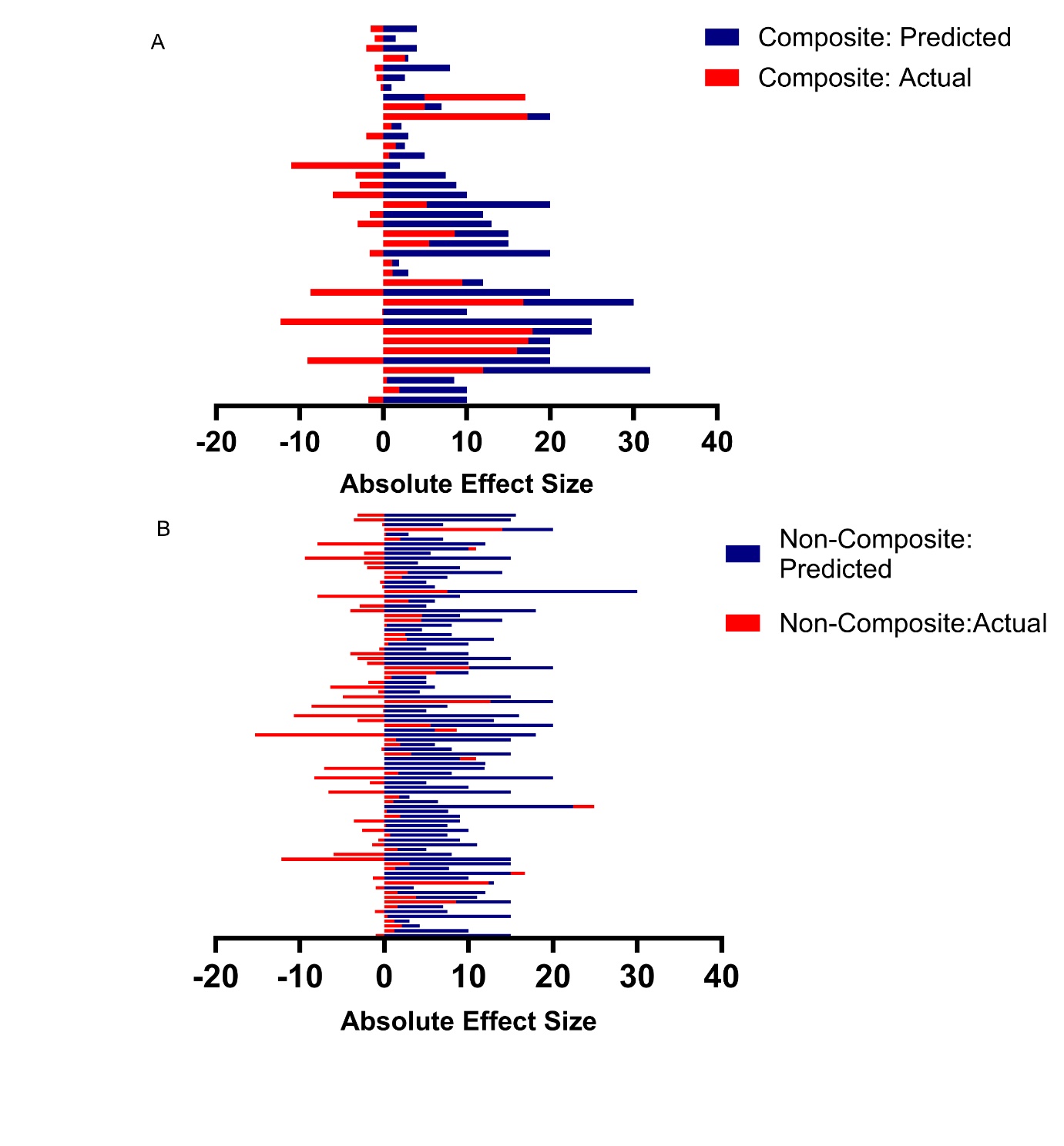
Event Rate Gap**

The event rate gap was calculated as: predicted event rate minus actual event rate in control group ^1^. Positive values indicate that the event rate was lower than predicted, and 0 would be that the achieved event rate in the control is exactly as predicted.

Continuous COMs were not included in this analysis due to the frequent differences in data presentation (e.g. mean quoted for sample size calculation and median presented in results).

Table S3 showing median event rate gap

|  | **Non-COM**  **(n=90)** | **Categorical COM**  **(n=23)** |
| --- | --- | --- |
| **Median Event Rate Gap (IQR)** | -1.9 (-9.5 – 4.3) | 0.9 (-5.8 – 6.6) |
| p = 0.40 | | |

Figure S6 showing median event rate gaps


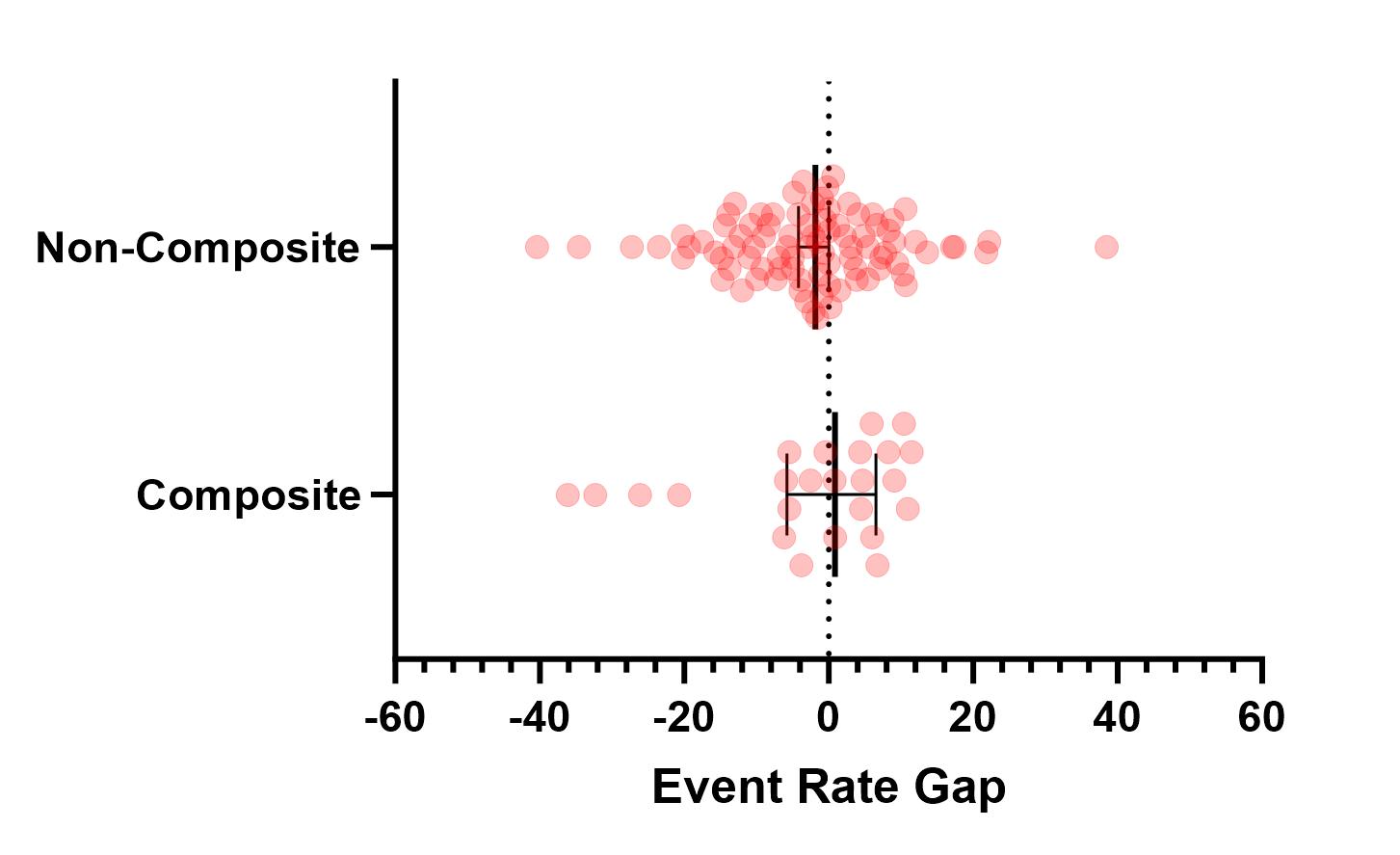


**Median predicted relative effect estimates**

Table S4 showing median predicted relative effect estimates.

|  | **Categorical COM (n = 26)** | **Continuous COM (n = 14)** | **Non-COM "Mortality” (n = 78)** | **Non-COM “Other” (n = 12)** |
| --- | --- | --- | --- | --- |
| **Median Predicted Relative Effect Estimates (IQR)** | 35.4 (24.8 – 50.0) | 20.2 (16.7 – 36.7) | 25.0 (19.6 – 32.7) | 38.9 (22.5 – 56.8) |

**Sensitivity Analyses**

The sensitivity analyes conducted included (i) including the ordinal outcomes as a COM, (ii) including the ordinal outcomes as a non-COM, (iii) removing the ordinal outcomes from the analysis all together.

When the ordinal outcomes are included as a COM the finding of increasing use remains significant when assessed with the Mann-Kendall test (p-value for trend < 0.001).

Table S5 including all ordinal scales as COM

|  | **COM (n = 92)** | **Non-COM (n = 102)** | **p – value** |
| --- | --- | --- | --- |
| **Median Number of Sites, n (IQR)** | 12 (3 – 35) | 31 (18 – 51) | <0.001 |
| **Median Number of Patients, n (IQR)** | 385 (200 – 740) | 748 (344 – 1880) | <0.001 |
| **Trial Power, % (IQR)** | 80 (80 – 85) | 80 (80 – 90) | 0.17 |
| **Statistically Significant, n (%)** | 19 (20.7%) | 17 (16.7%) | 0.58 |

Table S6 including all ordinal scales as non COM

|  | **COM (n = 73)** | **Non-COM (n = 121)** | **p - value** |
| --- | --- | --- | --- |
| **Median Number of Sites, n (IQR)** | 14 (4 – 36) | 29 (11 – 39) | 0.005 |
| **Median Number of Patients, n (IQR)** | 400 (198 – 868) | 600 (302 – 1372) | 0.002 |
| **Trial Power, % (IQR)** | 80 (80 – 85) | 80 (80 – 90) | 0.035 |
| **Statistically Significant, n (%)** | 15 (20.5) | 21/121(17.4) | 0.57 |

Table S7 excluding ordinal outcomes from the analysis all together

|  | **COM (n = 73)** | **Non-COM (n = 102)** | **p - value** |
| --- | --- | --- | --- |
| **Median Number of Sites, n (IQR)** | 14 (4 – 36) | 31 (18 – 51) | < 0.001 |
| **Median Number of Patients, n (IQR)** | 400 (198 – 868) | 748 (344 – 1880) | < 0.001 |
| **Trial Power, % (IQR)** | 80 (80 – 85) | 80 (80 – 90) | 0.022 |
| **Statistically Significant, n (%)** | 15 (20.5) | 17 (16.7) | 0.56 |

Table S8 comparing ordinal outcomes against COM

|  | **COM (n = 73)** | **Ordinal (n = 19)** | **p - value** |
| --- | --- | --- | --- |
| **Median Number of Sites, n (IQR)** | 14 (4 – 36) | 11 (3 – 26) | 0.72 |
| **Median Number of Patients, n (IQR)** | 400 (198 – 868) | 370 (210 – 506) | 0.45 |
| **Trial Power, % (IQR)** | 80 (80 – 85) | 80 (80 – 82 | 0.73 |
| **Statistically Significant, n (%)** | 15 (20.5) | 4 (21.1) | 1.00 |
| **Manuscripts in which outcome is stated as a COM, n (%)** | 37 (50.7) | 3 (15.8%) | 0.007 |
| **Each component presented separately in the table of results, n (%)** | 48 (65.8) | 7 (36.8%) | 0.03 |

**Ordinal Outcomes**

Of the 19 ordinal outcomes 7 (36.8%) reported CPC (cerebral performance category), 6 (31.2%) reported the GOS (-E) (Glasgow Outcome Scale [Extended]), 3 reported (15.8%) mRS (modified Rankin Scale) and 3 reported (15.8%) the WHO clinical progression scale for COVID-19. With respect to the presentation and analysis of these scales:

- 7 (36.8%) presented each individual level of the ordinal scale in the table of results (or with clear proportions of the number of patients in each level within a figure in the main manuscript).
- 10 (52.6%) of the studies provided a graphical representation of the ordinal scale breakdown, however these figures did not include the specific numbers of participants in each category.
- 14 (73.7%) of the ordinal outcomes were dichotomised. Of these, 10 (71.4%) reported the favourable aspect as the primary outcome (e.g. survival with a good neurological outcome).

Table S9 showing the breakdown between favourable and unfavourable outcomes when ordinal outcomes were dichotomised

| **Outcome** | **Dichotomisation Cut-off** | **Number of trials using cut-off** |
| --- | --- | --- |
| **CPC** | Favourable Outcome ≤ 2 | 7 |
| **GOS / GOS – E** | Favourable Outcome = 5 | 4 |
| **mRS** | Favourable Outcome ≤ 3 | 2 |
|  | Favourable Outcome ≤ 4 | 1 |
| **WHO Covid Scale** | Improvement defined as reduction by ≥ 2 levels in scale | 3 |

**References**

1 - Summers MJ, Chapple LA, McClave SA, Deane AM. Event-rate and delta inflation when evaluating mortality as a primary outcome from randomized controlled trials of nutritional interventions during critical illness: a systematic review. *Am J Clin Nutr*. 2016;103(4):1083-1090
